# Mechanism, kinetics and selectivity of selenocyclization of 5-alkenylhydantoins: an experimental and computational study

**DOI:** 10.3762/bjoc.11.200

**Published:** 2015-10-07

**Authors:** Biljana M Šmit, Radoslav Z Pavlović, Dejan A Milenković, Zoran S Marković

**Affiliations:** 1 Faculty of Science, University of Kragujevac, Radoja Domanovića 12 P.O. Box 60, 34000 Kragujevac, Serbia; 2 Bioengineering Research and Development Center, 34000 Kragujevac, Serbia; 3 Department of Chemical-Technological Sciences, State University of Novi Pazar, Vuka Karadžića bb, 36300 Novi Pazar, Serbia

**Keywords:** density functional theory, fused bicyclic hydantoins, intermediate, reaction mechanism, regioselectivity, selenocyclization

## Abstract

The mechanism and selectivity of a bicyclic hydantoin formation by selenium-induced cyclization are investigated. The proposed mechanism involves the intermediates formed by an electrophilic addition of the selenium reagent on a double bond of the starting 5-alkenylhydantoin prior the cyclization. These intermediates are readily converted into the more stable cyclic seleniranium cations. A key step of the mechanism is an intramolecular cyclization which is realized through an *anti*-attack of the internal nucleophile, the amidic nitrogen, to the seleniranium cation yielding the intermediate imidazolinium cations. Their deprotonation is followed by the formation of the fused bicyclic reaction products. Important intermediates and key transition states are studied by using density functional theory (DFT) methods. The pathways of the reaction are investigated in detail. There are two regioselective pathways related to 5-*exo* and 6-*endo* products. Theoretical calculations and the monitoring of the cyclization reaction using ^1^H NMR spectroscopy are in a good agreement with the proposed mechanism and are consistent with our experimental results. The preferred pathway for formation of 5-*exo* products is confirmed.

## Introduction

Hydantoins, a class of heterocyclic compounds with cyclic urea core, are widely used as intermediates in industrial production of α-amino acids [1]. They exhibit various biological activities making them attractive candidates for drug discovery [2–11]. In the most cases, the observed biological activities do not arise from the hydantoin nucleus itself but from different substituents that have been attached to it. During the last decades, the effect of the structural modification on the biological activity of the hydantoin derivatives has been studied intensively [12]. Due to their various biological activities polycyclic hydantoins, especially spirohydantoins [13–20] and fused [21–23] bicyclic hydantoin derivatives, have recently attracted great interest in both organic and medicinal chemistry.

In our previous work, we reported the experimental results on the selenocyclization of 5-alkenylhydantoins leading to fused bicyclic products [24]. To our best knowledge, no previous theoretical work has reported on this reaction. In this paper, density functional theory (DFT) [25–28] is utilized to understand the mechanism and selectivity of this key step in the synthesis of fused bicyclic hydantoins. ^1^H NMR spectroscopy monitoring, as well as ^1^H NMR chemical shifts prediction [29–34] is used as a useful tool in the search of the most probable intermediates in the reaction. The theoretical results are discussed and compared with our experimental observations.

## Results and Discussion

We have recently reported a three-step reaction sequence to *cis*-fused bicyclic hydantoins involving selenium-induced cyclization of 5-alkenylhydantoins as the key step [24]. The reaction is chemo- and regiospecific giving only the five-membered bicyclic hydantoins, 5-*exo* products, in good to excellent yields (56–96%). The cyclization is stereoselective obtaining a separable diastereomeric mixture, and the products with the bridgehead substituent and the CH_2_SePh group in *cis* relationship are formed predominately.

**Scheme 1:**
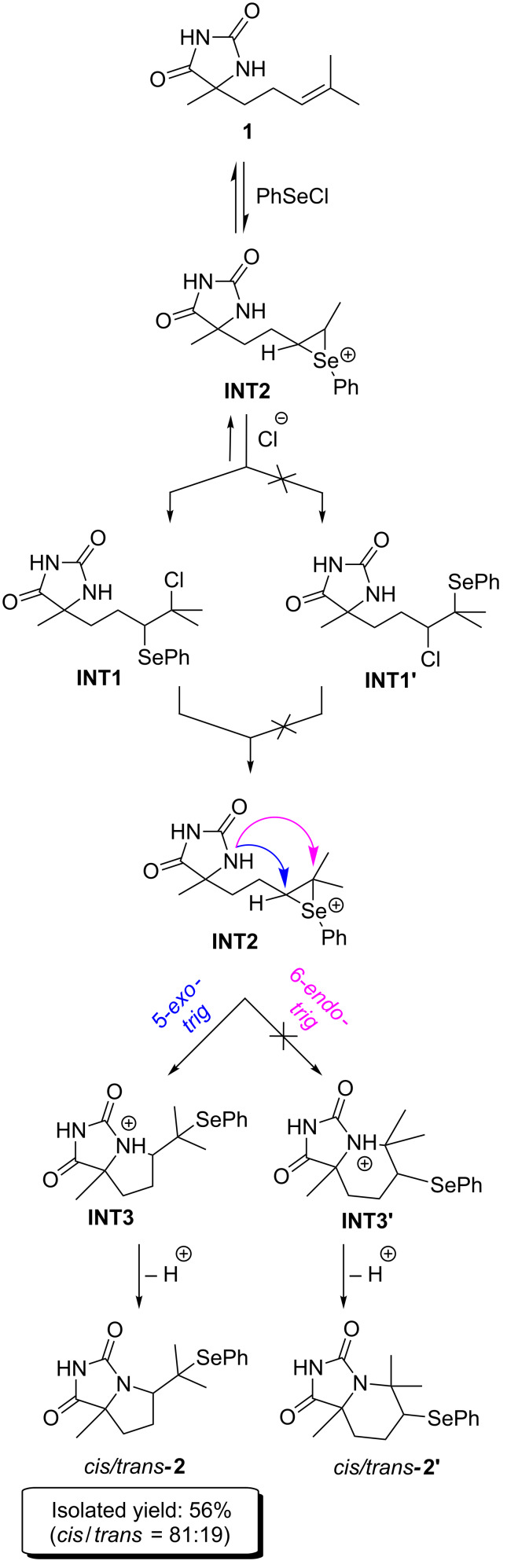
Proposed mechanism for the selenocyclization of model substrate **1**.

**Scheme 2:**
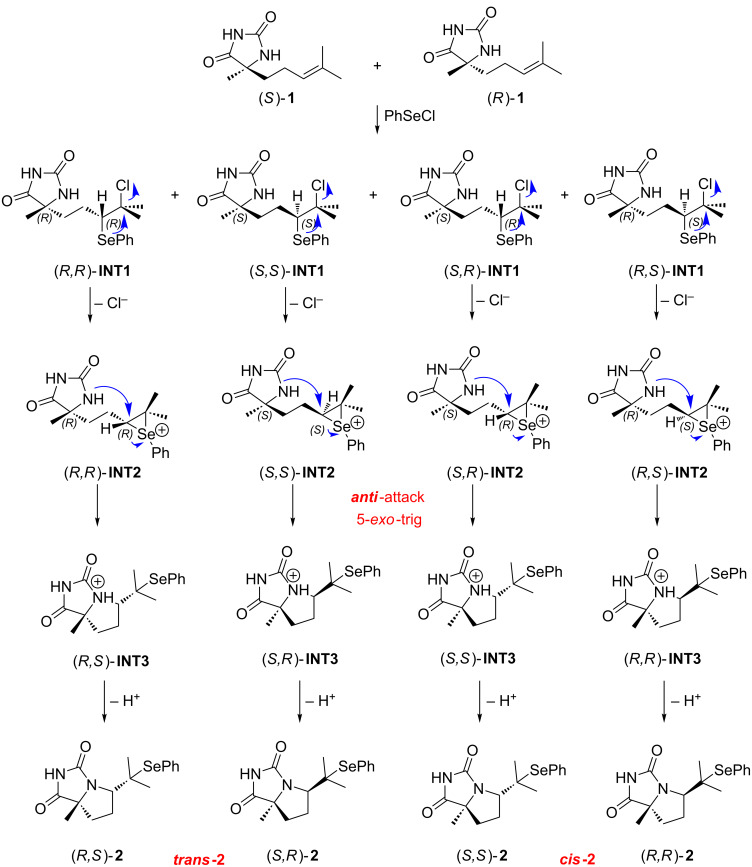
5-*Exo* pathway of the proposed mechanism with all possible intermediates and products.

To gain deeper insight into the cyclization process leading to the formation of fused bicyclic hydantoins, a computational (DFT) study was carried out. The mechanism of the reaction of 5-alkenylhydantoins with phenylselenyl chloride was examined using the reaction with model substrate **1**. The proposed five-step mechanism, starting with an electrophilic addition of PhSeCl to the double bond, is presented in Scheme 1, while the 5-*exo* pathway with all stereoisomers is presented in Scheme 2. The first step of the proposed mechanism producing the seleniranium cation, followed by *anti-*attack of the external nucleophile (Cl^−^) to the double bond [35] of 5-alkenylhydantoin is supposed to be reversible, and leads to the formation of two theoretically possible pairs of regioisomeric intermediates, (*S*,*R*/*S*,*S*)**-INT1** (Markovnikov products) and (*S*,*R*/*S*,*S*)**-INT1’** (*anti*-Markovnikov products) (Scheme 1). A similar mechanism was proposed earlier for cyclofunctionalization of olefinic urethanes [36].

**Figure 1:**
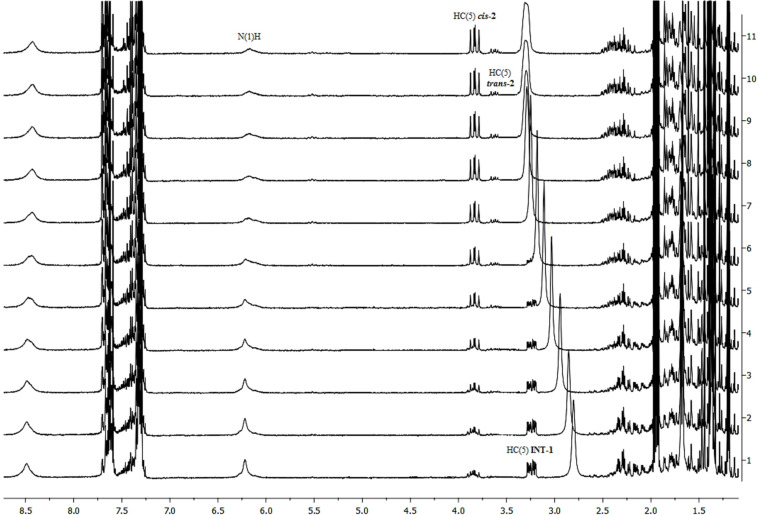
^1^H NMR monitoring of the cyclization of 5-alkenylhydantoin **1** with PhSeCl in acetonitrile-*d*_3_ solution in the presence of silica gel at ambient temperature during 15 h.

The formation of addition products as intermediates in the second step was proposed after monitoring of the reaction by using ^1^H NMR spectroscopy (Figure 1). Also, in this experiment a regioselective formation of Markovnikov-type products of the addition of selenium reagent to the double bond was noticed. To further verify the nature of these intermediates we have computed ^1^H chemical shifts by means of DFT GIAO computations.

In the first spectrum of time-dependent experiment, a signal corresponding to the olefinic proton of starting 5-alkenylhydantoin, triplet of septets at 5.04 ppm (Supporting Information File 1, Figure S1), disappeared completely, while the characteristic signal of the C(5)H proton (see Table 1 for label assignments) of the product **2**, doublet of doublets at 3.83 ppm began to appear. This observation prompts us to propose the above mentioned mechanism which includes the product of the addition of PhSeCl on a double bond as intermediate in the cyclization process (Scheme 1). Formation of this intermediate is obviously too fast for NMR time scale. The new signal at 3.24 ppm (Figure 1) which is not corresponding neither to substrate nor product additionally sustains the statement about existing of such an intermediate. Theoretical calculations of the ^1^H chemical shifts for intermediate (*S*,*R*/*S*,*S*)*-***INT1** are in agreement with the observed values (Table 1, Figure 1).

**Table 1:** Calculated and experimental ^1^H chemical shifts of selected protons.^a^

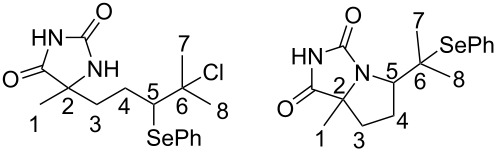							
	(*S*,*R*)-**INT1**	(*S*,*S*)-**INT1**	**INT1**	(*S*,*R*)-**2**		(*S*,*S*)-**2**	

	Calcd	Calcd	Exp^b^	Calcd	Exp	Calcd	Exp

C(5)H	3.6	3.6	3.3	3.7	3.5	4.1	3.9
C(3)H_2_	2.5	2.1	2.2	2.2	2.4	2.6	2.4
C(4)H_2_	2.0	2.2	2.3	2.0	1.9	1.9	1.9
C(1)H_3_	2.2	1.8	1.7	1.8	1.7	1.5	1.5
C(7)H_3_	1.4	1.4	1.4	1.3	1.5	1.4	1.4
C(8)H_3_	1.5	1.7	1.4	1.7	1.7	1.4	1.4
Mean absolute error (MAE)	0.25	0.15		0.13		0.10	
δ_(exp)_ = *a* + bδ_(calc)_	*a* = 0.19, *b* = 0.85	*a* = 0.11, *b* = 0.91		*a* = 0.26, *b* = 0.88		*a* = 0.09, *b* = 0.92	
S	0.28	0.19		0.15		0.05	
F	28.96	68.96		120.2		1654.62	
N	6	6		6		6	
R	0.940	0.970		0.980		0.999	
R^2^	0.880	0.940		0.970		0.998	

^a^Numeration of C-atoms given in the structures are for calculation and comparison with experimental data only. ^b^Experimental values are given at nearest 0.1 ppm.

Starting 5-alkenylhydantoin **1** was synthesized from 6-methyl-hepten-2-on [24] by Bucherer–Bergs reaction which is not stereoselective and thus obtained as racemic mixture. All calculations were done for the enantiomers with *S* configuration at the quaternary carbon atom of the hydantoin nucleus. The selected ^1^H chemical shifts of these intermediates, calculated and experimental, are listed in Table 1 while correlation plots are given in Supporting Information File 1, Figure S2.

**Figure 2:**
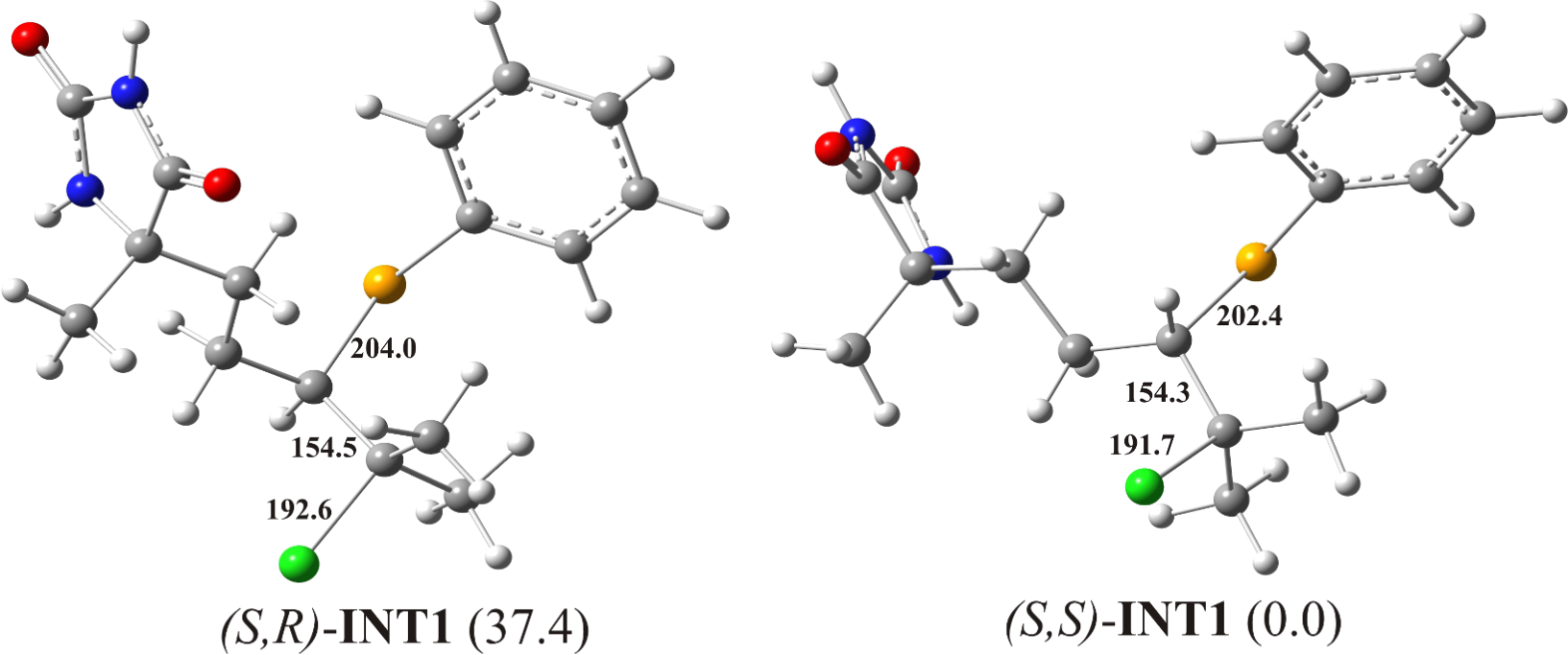
Optimized geometries of possible Markovnikov-type intermediates formed by the *anti-*stereospecific addition of PhSeCl on model substrate **1**, with relative free energy values indicated in kJ/mol. The crucial bond lengths are given in pm.

**Figure 3:**
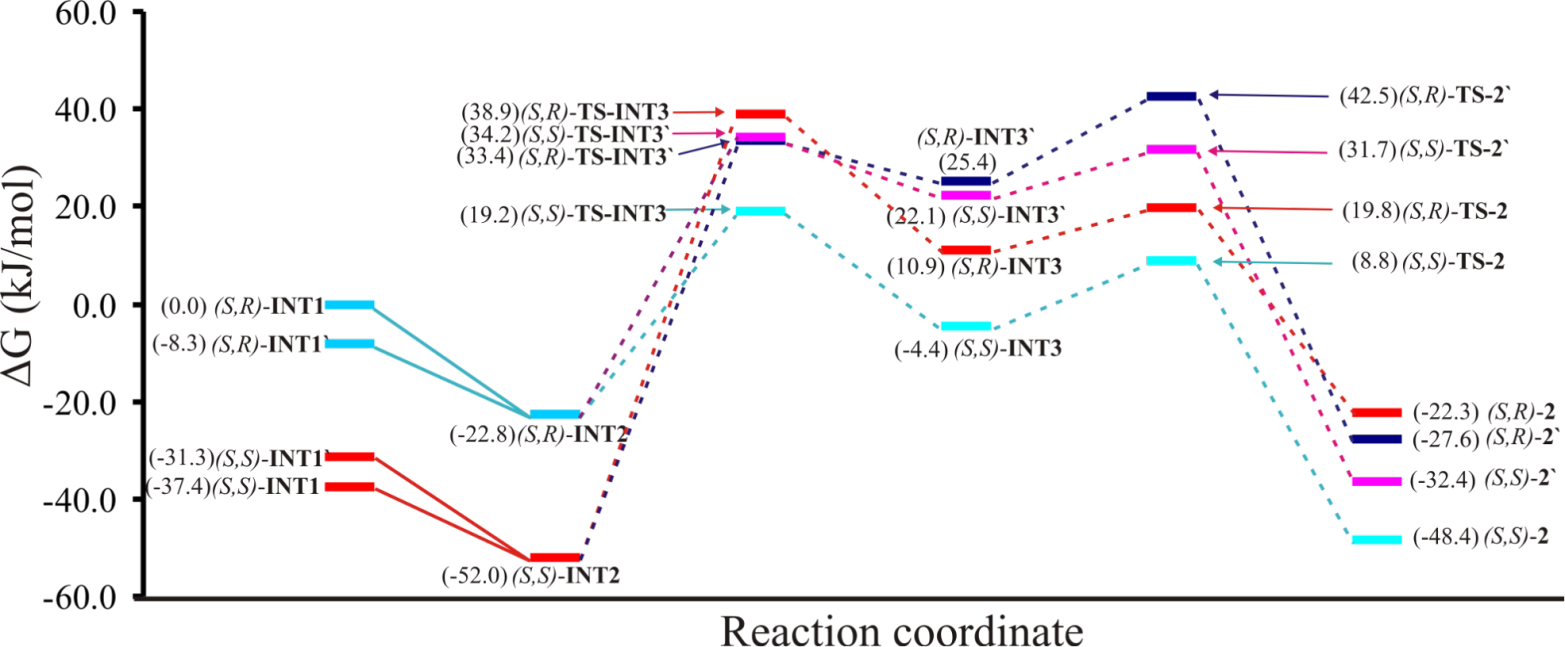
Energy profile for the proposed mechanism of selenocyclization of model substrate **1.** Relative energies are given in kJ/mol.

The simulated chemical shifts for the C(5)H proton of (*S*,*R*)-**INT1** and (*S*,*S*)-**INT1** are both 3.6 ppm, which correspond to the value of 3.3 ppm in the experimental spectra (Figure 1), whereas the chemical shifts for the C(5)H proton of (*S*,*R*)-**INT1’** and (*S*,*S*)-**INT1’** are notably larger (5.0 and 4.8 ppm, respectively) (Supporting Information File 1, Table S1). This finding indicates that the (*S*,*R*/*S*,*S*)-**INT1** (Markovnikov aduct) is formed as an intermediate of the examined reaction. On the basis of the calculated ^1^H chemical shifts (Supporting Information File 1, Table S1) it was possible to distinguish a configuration of these isomers, because calculated ^1^H chemical shifts for (*S*,*S*)-**INT1** had a better correlation with the experimental values. Moreover, calculations of relative free energies of these intermediates showed that the *S*-isomer is more stable (Figure 2 and Figure 3).

**Figure 4:**
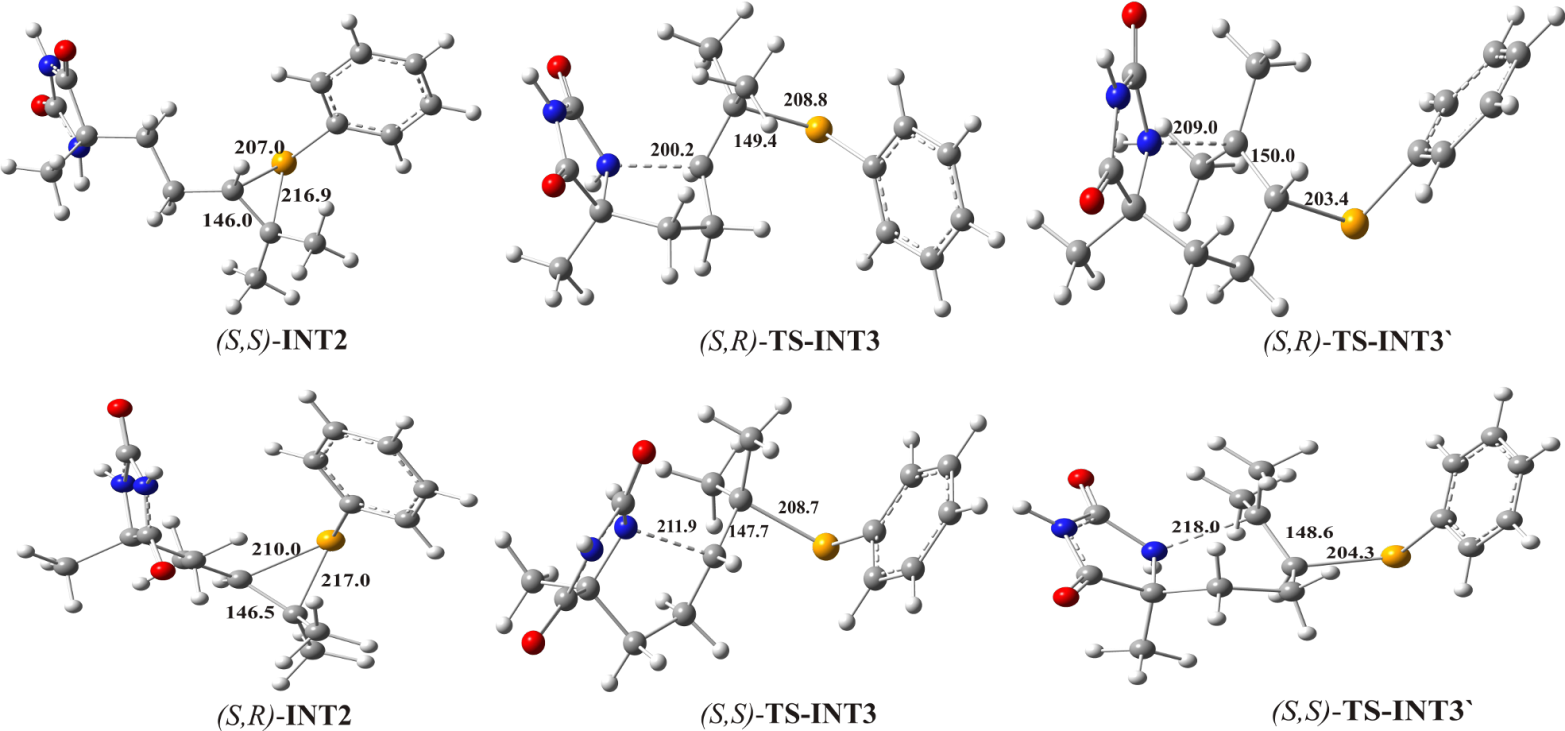
Optimized geometries for seleniranium cations and corresponding transition states for the formation of the five-membered ring (*S*,*R*)-**TS-INT3** and (*S*,*S*)-**TS-INT3** and six-membered ring (*S*,*R*)-**TS-INT3’** and (*S*,*S*)-**TS-INT3’**. The crucial bond lengths are given in pm.

In the third step, these intermediates are readily converted into the more stable cyclic seleniranium cations (*S*,*R*)-**INT2** and (*S*,*S*)-**INT2**. Calculations showed that (*S*,*S*)-**INT2** is significantly more stable (by about 30 kJ/mol) than (*S*,*R*)-**INT2** (Figure 3 and Figure 4). These intermediates are actually the reactant complexes for intramolecular cyclization.

**Figure 5:**
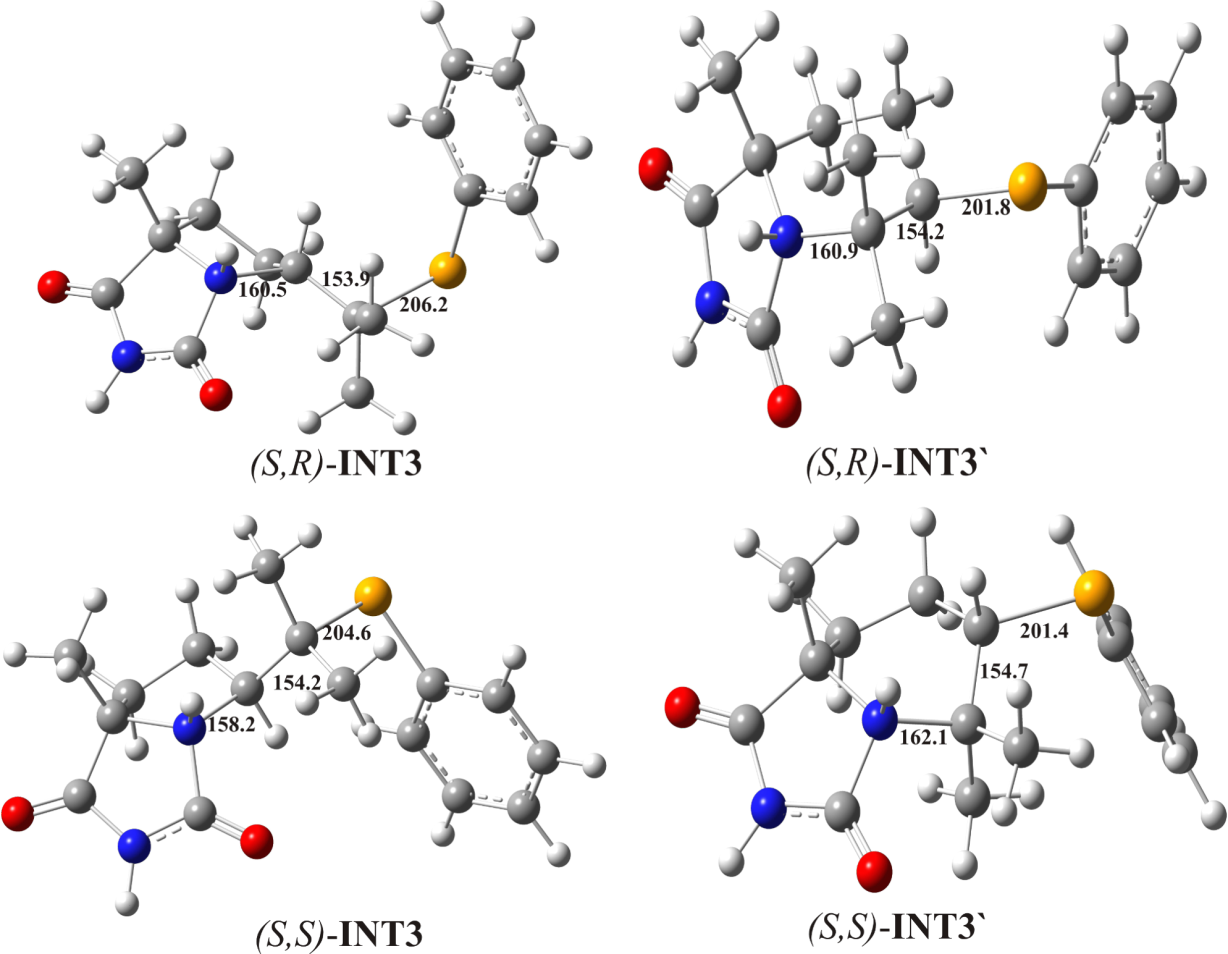
Optimized geometries of intermediate bicyclic imidazolinium cations. The crucial bond lengths are given in pm.

The fourth step of the proposed mechanism is a ring forming reaction which is achieved through an *anti-*attack of the internal nucleophile, amidic nitrogen N(1) at carbon C(5) or C(6) yielding the five-membered ((*S*,*R*)-**INT3** and (*S*,*S*)-**INT3**) or six-membered ((*S*,*R*)-**INT3’** and (*S*,*S*)-**INT3’**) intermediate bicyclic imidazolinium cations. Energy profiles for both 5-*exo* and 6-*endo* pathways are presented in Figure 3. The ring closure reactions are carried out via four transition states: two for the formation of the five-membered ring (*S*,*R*)-**TS-INT3** and (*S*,*R*)-**TS-INT3**, and two for the formation of the six-membered ring (*S*,*R*)-**TS-INT3’** and (*S*,*S*)-**TS-INT3’** (Figure 4). In (*S*,*R*)-**TS-INT3** and (*S*,*S*)-**TS-INT3** the N-C(5) bond is partially formed, the Se-C(5) bond is partially cleaved, whereas the Se-C(6) bond is becoming significantly shorter in comparison to (*S*,*S*)-**TS-INT3**. Similarly, in (*S*,*R*)-**TS-INT3’** and (*S*,*S*)-**TS-INT3’** the simultaneous cleavage of the Se-C(6) and formation of the N-C(6) bond, and shortening of the Se-C(5) bond occur. In the intermediates **INT3** the N-C(5) and Se-C(6) bonds, whereas in the intermediates **INT3’** the N-C(6) and Se-C(5) are almost completely formed. Optimized structures of seleniranium cations and transition states are given in Figure 4, while those for imidazolinium cations are given in Figure 5.

From the charge analysis (data given in Supporting Information File 1, Table S2) it is possible to deduce that the C(6) atom is more electrophilic than C(5) due to the positive inductive effect of the two methyl groups. However, on the other hand these methyl groups hinder the approach of the nucleophilic nitrogen N(1) to C(6). The selenium ion binds to the C(5) atom, which becomes positively charged and susceptible to the nucleophilic attack of N(1) atom. Subsequent ring closure is the rate-determing step and also the step in which the cyclization mode and distribution of the products is determined (Figure 3). This process was followed in ^1^H NMR experiments by the increase of the signal intensities of C(5)H protons of the products *cis-***2** and *trans*-**2** which is commensurate with the decrease of the signal intensities of C(5)H as well as N(1)H protons of the intermediate **INT-1** (Figure 1 and Table 1). It is well known that oxygen could compete with nitrogen as internal nucleophile leading to different heterocycles [37–38]. Disappearing of the signal of N(1)H is a clear proof that the amidic nitrogen from the hydantoin ring is an internal nucleophile.

**Figure 6:**
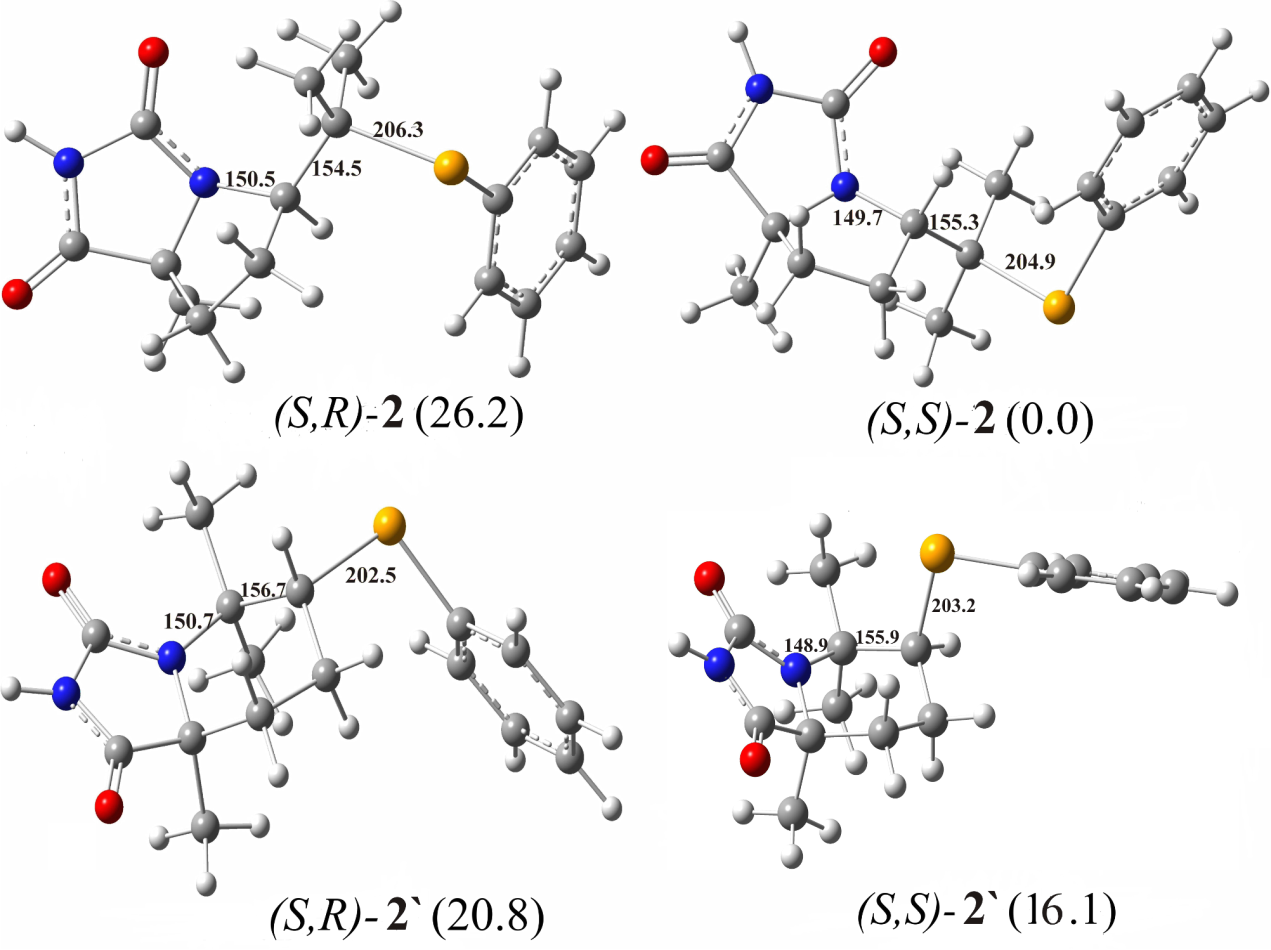
Optimized geometries of possible products of the selenocyclization of **1**, with relative free energy values indicated in kJ/mol. The crucial bond lengths are given in pm.

The last step of the reaction mechanism is the deprotonation of the intermediate imidazolinium cations by the Cl**^−^** anion followed by the formation of the fused bicyclic products. The calculated geometries and relative free energies of products are depicted in Figure 6. It is obvious that the products **2’** are less stable than (*S*,*S*)-**2** due to the repulsive 1,3-diaxial interactions between the two methyl groups. The simulated chemical shifts for C(5)H proton of (*S*,*R*)-**2** and (*S*,*S*)-**2** have excellent correlation to the experimental values (Table 1 and Supporting Information File 1, Figure S2).

Our investigations showed that the activation energies for the formation of (*S*,*R*)-**INT-3’** and (*S*,*R*)-**INT3** (*trans-*diastereoisomers) are mutually very similar, whereas that for the formation of (*S*,*S*)-**INT3’** and (*S*,*S*)-**INT3** (*cis-*diastereoisomers) are significantly lower (Table 2). But the free energy of both six-membered imidazolinium cations **INT3’** are very similar and match higher than those for five-membered **INT3.** The facts that six-membered imidazolinium cations **INT3’** are very unstable and that the activation energies for the inverse reactions are very low (about 10 kJ/mol) could be an explanation for the regioselective formation of five-membered bicyclic products **2**. On the basis of the calculated rate constants it is clear that the formation of (*S*,*S*)-**INT3** is most favorable.

**Table 2:** Calculated free activation energies (Δ*G*^≠^) and rate constants (*k*) for the formation of **INT3** and **INT3’** intermediates in acetonitrile.

Structure	Δ*G*^≠^ (kJ/mol)	*K* (M^−1^ s^−1^)

(*S*,*R*)-**INT3**	90.9	7.34 × 10^−4^
(*S*,*S*)-**INT3**	42.0	2.77 × 10^5^
(*S*,*R*)-**INT3’**	85.4	6.79 × 10^−3^
(*S*,*S*)-**INT3’**	57.0	6.52 × 10^2^

All parameters were calculated in acetonitrile as solvent.

All these findings show that the formation of (*S*,*S*)-**2** is both kinetically and thermodynamically favored, which is in agreement with the experimental results that (*S*,*S*)-**2** is the major product of the reaction [24]. The formation of (*S*,*R*)-**2** is thermodinamically controlled. A possible reason for the pronounced instability of (*S*,*R*)-**2** can be the destabilizing steric interaction between the hydantoin ring and the SePh group. Much lower formation of this product is in accordance with experimental results where the diastereomeric ratio *cis*/*trans* = 81:19 is obtained [24].

**Scheme 3:**
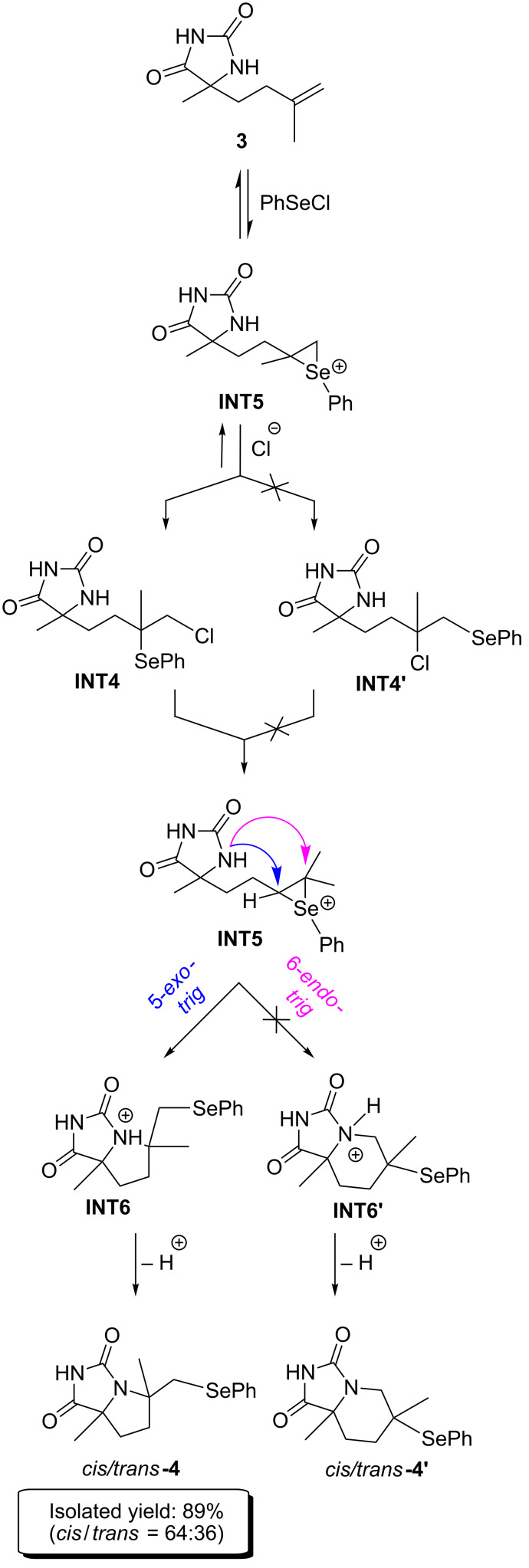
Proposed mechanism for selenocyclization of model substrate **3**.

With intent to verify the above proposed mechanism, we explored it on the other model substrate **3** with a different alkenyl moiety (Scheme 3).

**Figure 7:**
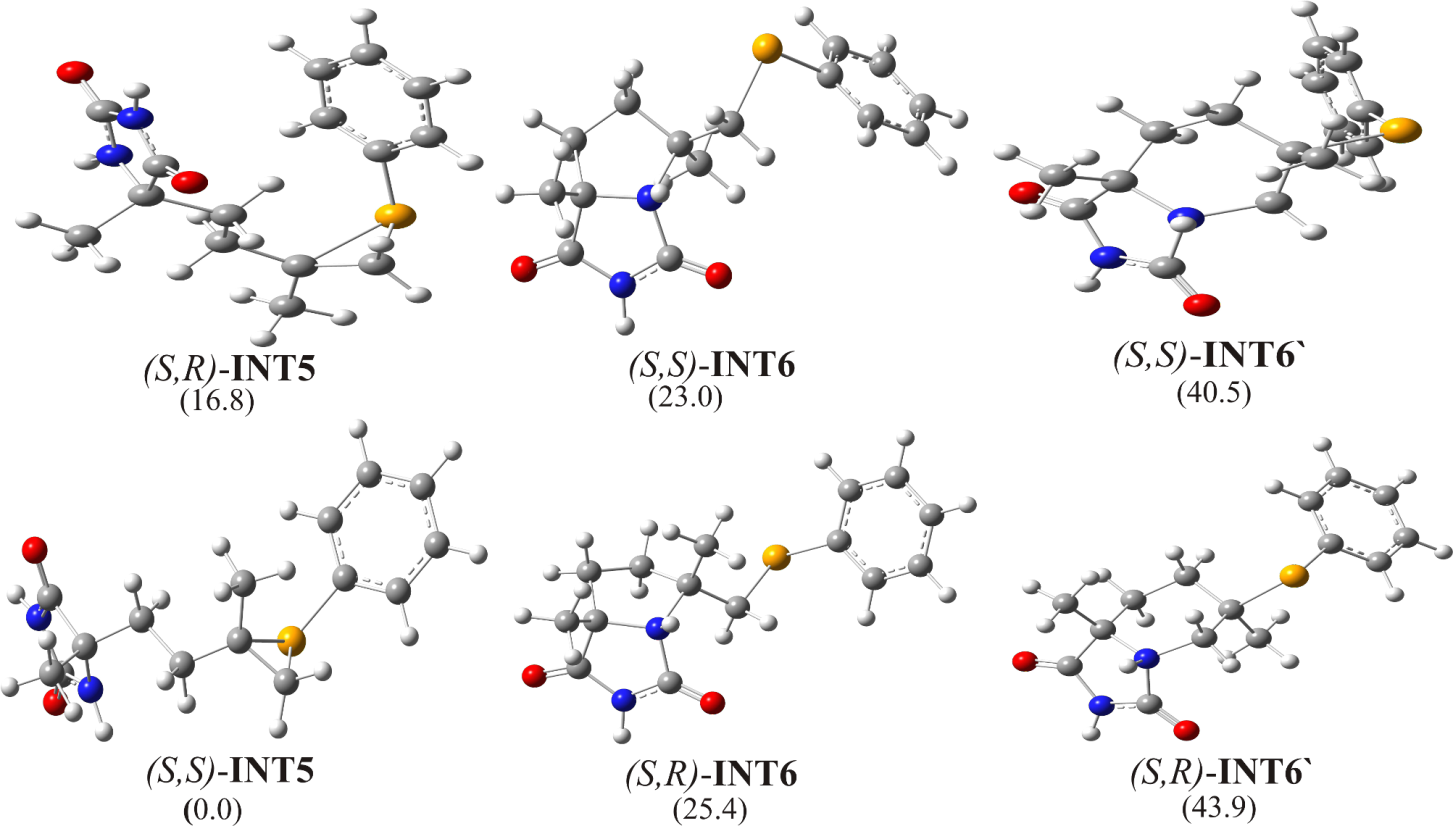
Optimized geometries of possible intermediates of the selenocyclization of model substrate **3**, with relative free energy values indicated in kJ/mol.

**Figure 8:**
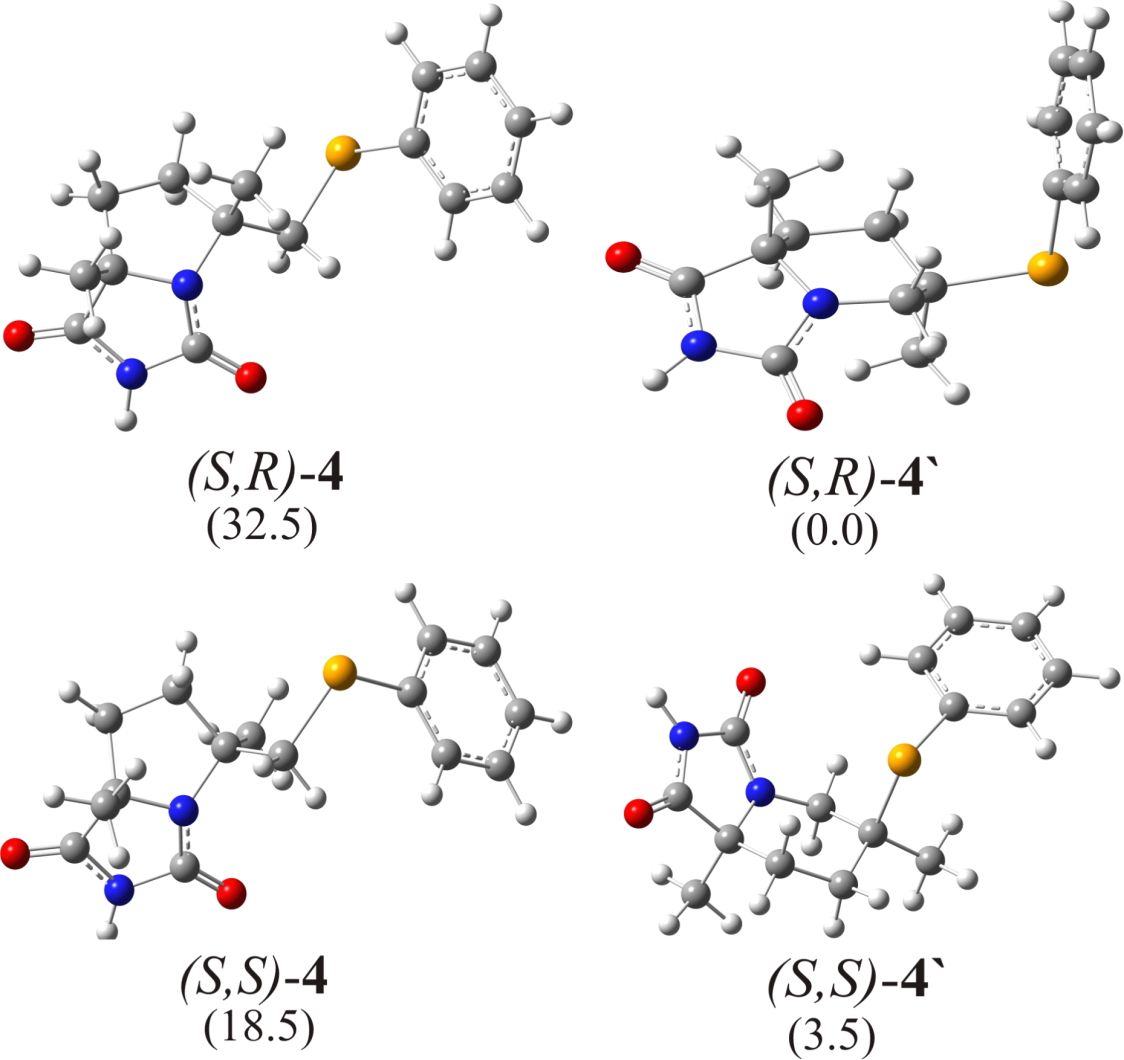
Optimized geometries of possible products of the selenocyclization of model substrate **3**, with relative free energy values indicated in kJ/mol.

The optimized structures of all possible intermediates are presented in Figure 7. From calculated free energies for all intermediates it could be concluded that the formation of five-membered products is favored again. Also, *cis*-diastereoisomers are more stable than *trans-*diastereoisomers, but there is no significant difference (Figure 8). It is in accordance with experimental results where diastereomeric ratio *cis*/*trans* = 64:36 is obtained [24].

## Conclusion

In summary, this work has provided the first theoretical study of the selenocyclization of 5-alkenylhydantoins. By ^1^H NMR monitoring of the reaction we proposed a five-step mechanism which involves the intermediate formed by the addition of selenium reagent on starting 5-alkenylhydantoin. The Markovnikov-type product is favored. These intermediates are readily converted into the more stable cyclic seleniranium cations. The key step is a selenium-induced intramolecular cyclization which is accomplished through an *anti*-attack of the internal nucleophile, amidic nitrogen, to the seleniranium cation yielding the intermediate imidazolinium cations. The last step of the reaction is deprotonation followed by the formation of the fused bicyclic hydantoins. DFT calculations support a plausible reaction pathway where the ring closure is the rate-determining step. The preferred 5-*exo* cyclization is confirmed. Elucidation of regio- and stereoisomerism of all intermediates and products were done on the basis of excellent correlation of experimental and calculated ^1^H chemical shifts as well as relative free energies. Theoretical calculations are in good agreement with our experimental results.

## Experimental

All commercials were used as received without further purification. Model compounds **1** and **3** and the corresponding products **2** and **4**, respectively, were synthesized according the reported procedures [24] and fully characterized by spectroscopic methods.

### ^1^H NMR measurements

^1^H NMR spectra were recorded on a 200 MHz NMR spectrometer in CD_3_CN as a solvent. Tetramethylsilane (δ = 0.0 ppm) was used as internal reference and chemical shifts are reported to the nearest 0.01 ppm.

^1^H NMR measurements of the cyclization reaction of hydantoin **1** (4.7 mg, 0.024 mmol, 1 equiv) with PhSeCl (5.0 mg, 0.026 mmol, 1.1 equiv) in the presence of SiO_2_ (7.2 mg, 0.119 mmol, 5 equiv) in acetonitrile-*d*_3_ (5 mL) were carried out in the standard 5 mm NMR tube. All spectra were recorded at ambient temperature over the period of 15 h.

### Computational methods

All calculations were conducted using Gaussian 09 [39] with the B3LYP functional [40–41] and the split-valence triple-zeta basis set 6-311G [42–43]. To attain better description of the delocalization effects that are crucial for the geometry and electronic structure of the investigated molecules, diffuse functions were added to the heavy atoms. The *p* and *d* polarization functions were also used. Full geometry optimizations, without any symmetry constraints, and frequency calculations were performed for all species in acetonitrile as solvent (dielectric constant = 35.688) using the SMD solvation model [44]. SMD is a continuum solvation model based on the quantum mechanical charge density of a solute molecule interacting with a continuum description of the solvent. Frequency calculations were performed to confirm that the optimized structures are energetic minima (no imaginary frequencies). A natural bond orbital (NBO) analysis [45–47] was performed for all species.

Relative free energies were calculated at *T* = 298 K. Rate constants were calculated using Transition state theory as implemented in TheRate program [48] and 1 M standard state as:


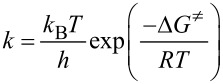


where *k*_B_, *h* and *R* stand for the Boltzman, Planck, and the gas constant, and Δ*G*^≠^ is the free activation energy, which is calculated as the G (Gibbs free energy) difference between transition states and reactants.

The ^1^H NMR spectra of the intermediates and products in acetonitrile were simulated. The geometry of TMS in acetonitrile was optimized using the B3LYP/6-311+G(d,p) and SMD models. We chose to use the B3LYP functional based on previously demonstrated good NMR performance for containing systems like those studied here [49]. The nuclear magnetic shielding tensors were calculated for TMS, intermediates and products using the Gaussian GIAO (gauge independent atomic orbital) method. The values for all hydrogen atoms in intermediates and products were subtracted from the value for hydrogen in TMS (31.91). All compounds belong to the *C*_1_ point group, and, thus, all hydrogens show different chemical shifts. For this reason the corresponding mean values were taken to represent the chemical shifts of the protons bonded to a certain carbon.

## Supporting Information

File 1 Copies of ^1^H NMR and ^13^C spectra of model substrates, additional Figures and Tables referred to the text, as well as Cartesian coordinates and total energies of all the stationary points discussed in the manuscript.
