# Supplementary material for: Mechanism, kinetics and selectivity of selenocyclization of 5-alkenylhydantoins: an experimental and computational study
Source: Beilstein J Org Chem. 2015 Oct 7;11:1865–75. doi: 10.3762/bjoc.11.200 (PMC4661018; doi:10.3762/bjoc.11.200)
Supplement: File 1 — Copies of 1H NMR and 13C spectra of model substrates, additional Figures and Tables referred to the text, as well as Cartesian coordinates and total energies of all the stationary points discussed in the manuscript. [file Beilstein_J_Org_Chem-11-1865-s001.pdf]

Supporting Information

for

Mechanism, kinetics and selectivity of selenocyclization of 5-alkenylhydantoins: an experimental and computational study

Biljana M. Šmit[*1], Radoslav Z. Pavlović[1], Dejan A. Milenković[2] and Zoran S. Marković[2,3]

Address: [1]Faculty of Science, University of Kragujevac, Radoja Domanovića 12 P.O. Box 60, 34000 Kragujevac, Serbia, [2]Bioengineering Research and Development Center, 34000 Kragujevac, Serbia and [3]Department of Chemical-Technological Sciences, State University of Novi Pazar, Vuka Karadžića bb, 36300 Novi Pazar, Serbia

Email: Biljana M. Šmit[*] - biljam@kg.ac.rs

[*]Corresponding author

Copies of ^{1}H NMR and ^{13}C spectra of model substrates, additional Figures and Tables referred to the text, as well as Cartesian coordinates and total energies of all the stationary points discussed in the manuscript.

Table of contents:

Figure S1: ^{1}H and ^{13}C NMR spectra of model substrate **1**. S1

Copies of ^{1}H and ^{13}C NMR spectra of all new compounds S2–S6

Figure S2: Correlation between calculated and experimental ^{1}H chemical shifts (ppm). S7

Table S1: Calculated proton chemical shifts for intermediates and products. S7

Table S2: Calculated NBO charges for seleniranium cations (*S*,*R*)-**INT2** and (*S*,*S*)-**INT2**. S8

Cartesian coordinates and electronic energies for all compounds. S8–S41

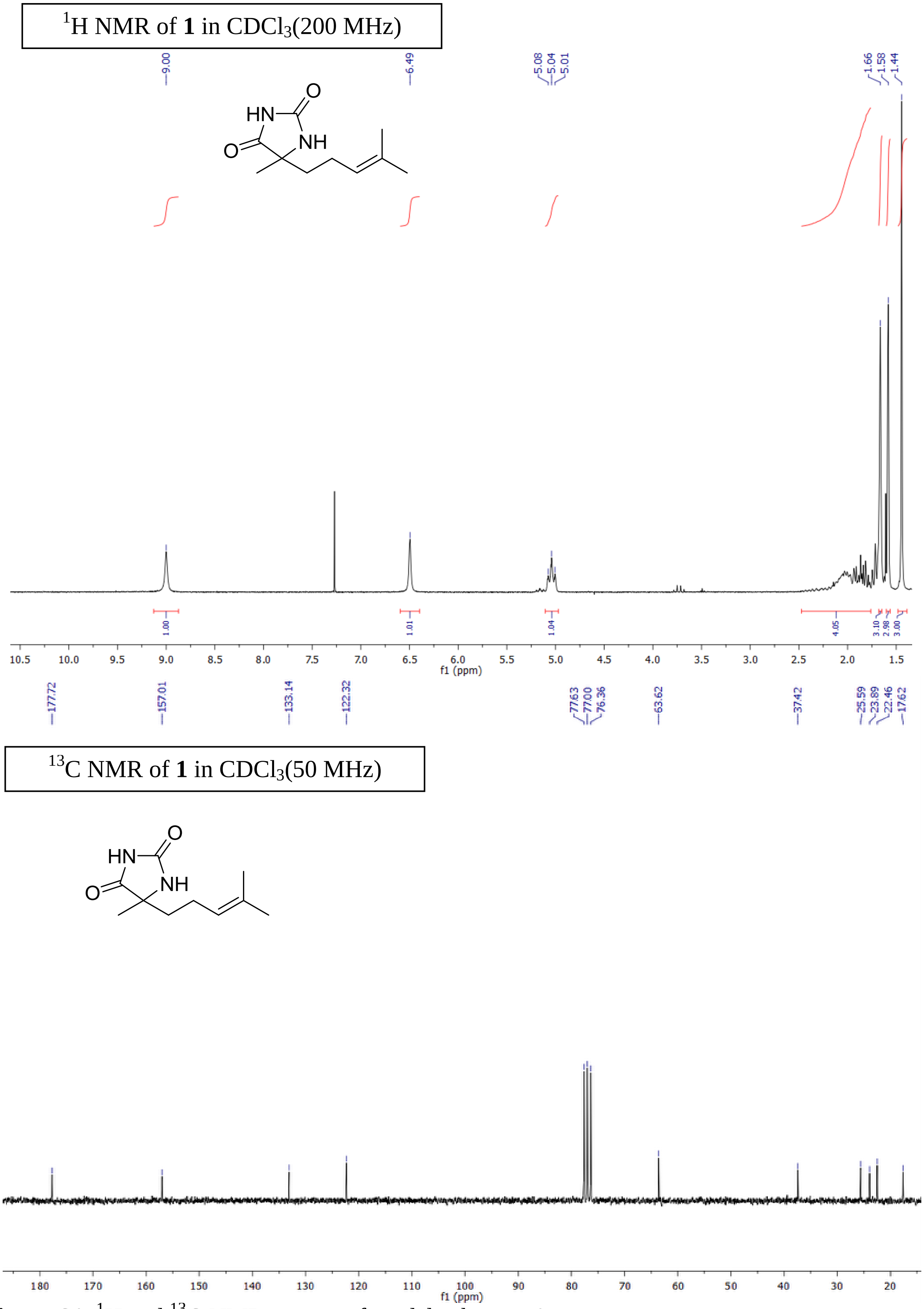

Figure S1. ^{1}H and ^{13}C NMR spectra of model substrate **1**

^{1}H NMR of ***cis*-2** in $CDCl_3$(200 MHz)

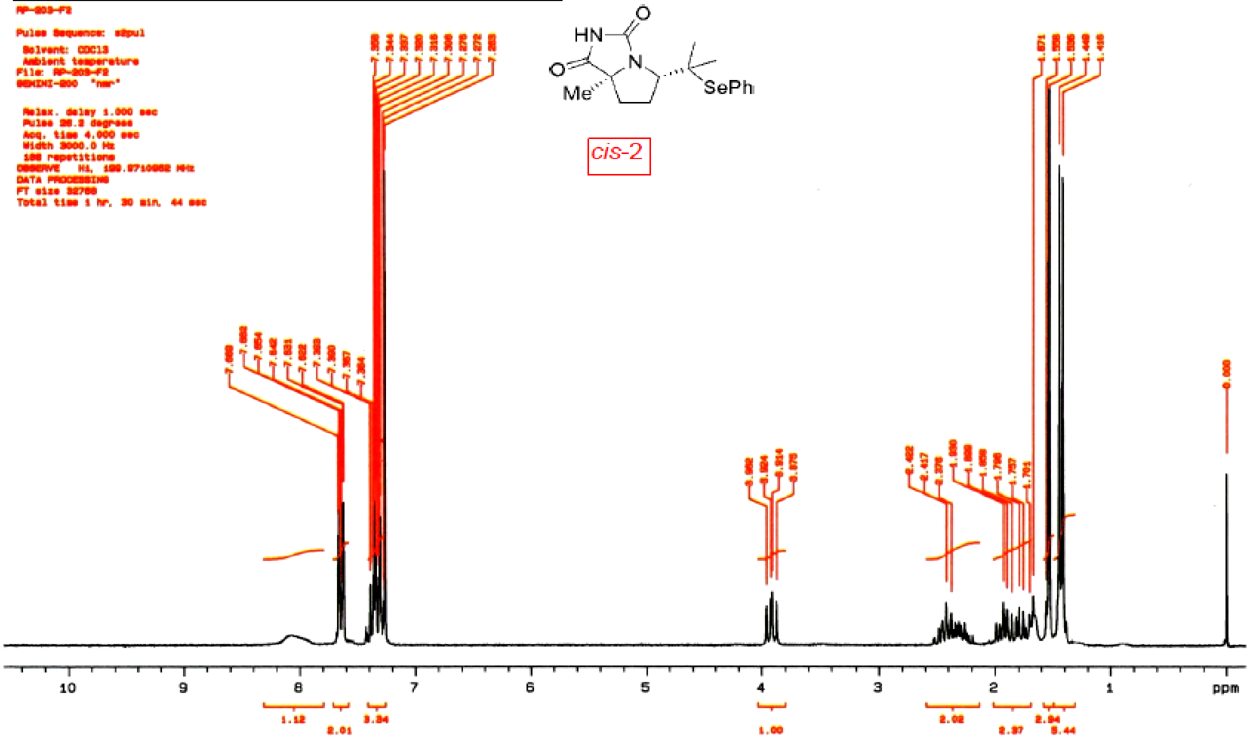

^{13}C NMR of ***cis*-2** in $CDCl_3$(50 MHz)

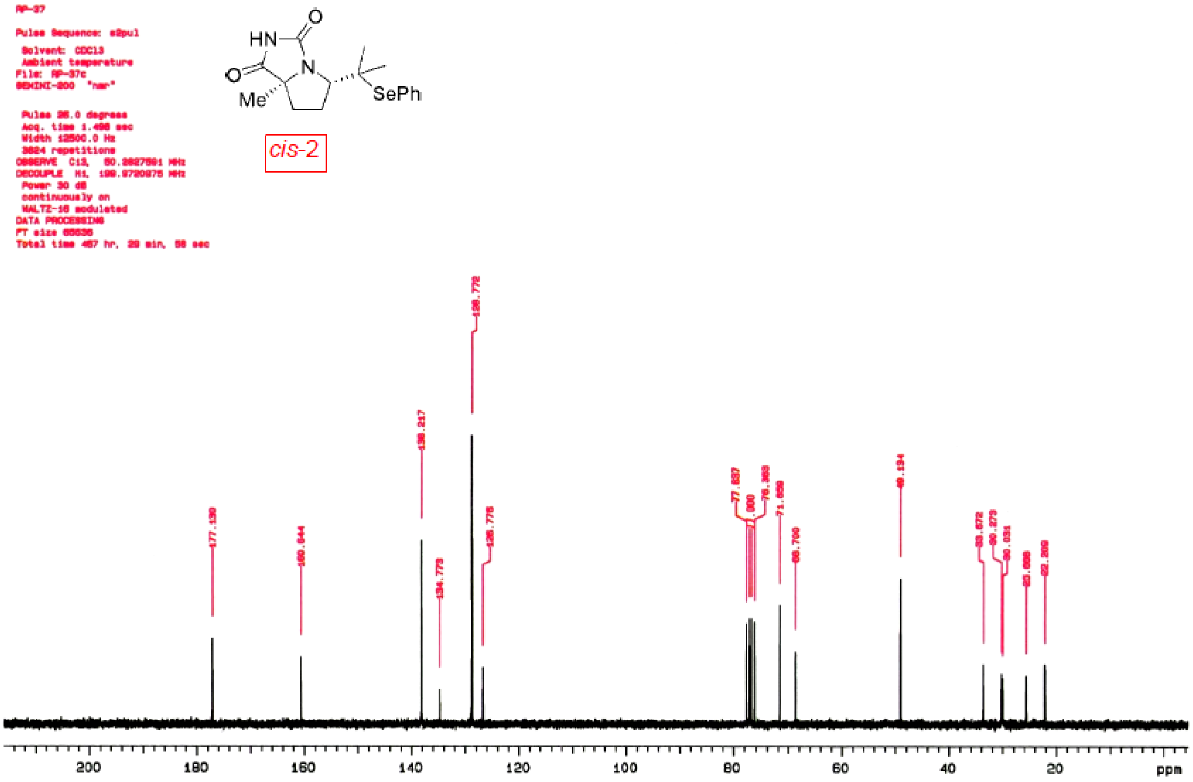

^{1}H NMR of ***trans*-2** in $CDCl_3$(500 MHz)

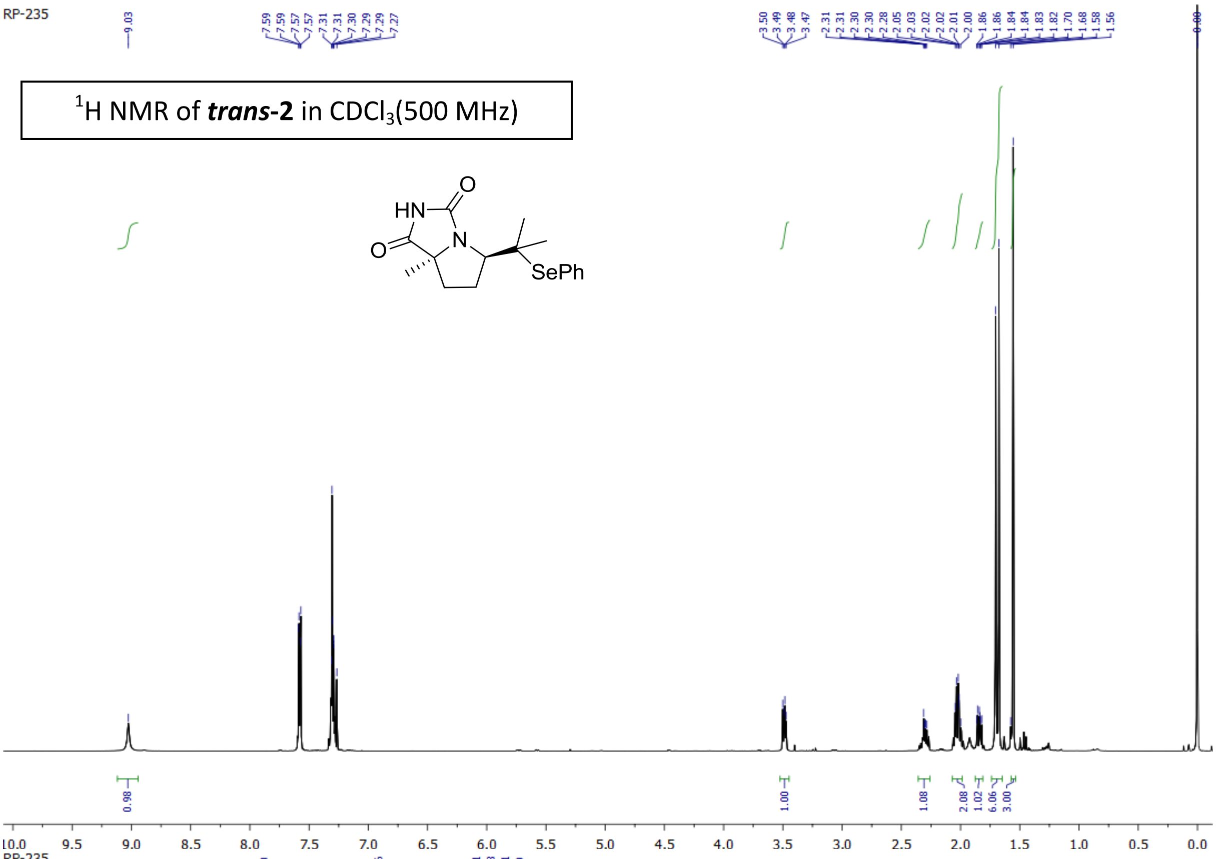

^{13}C NMR of ***trans*-2** in $CDCl_3$(125 MHz)

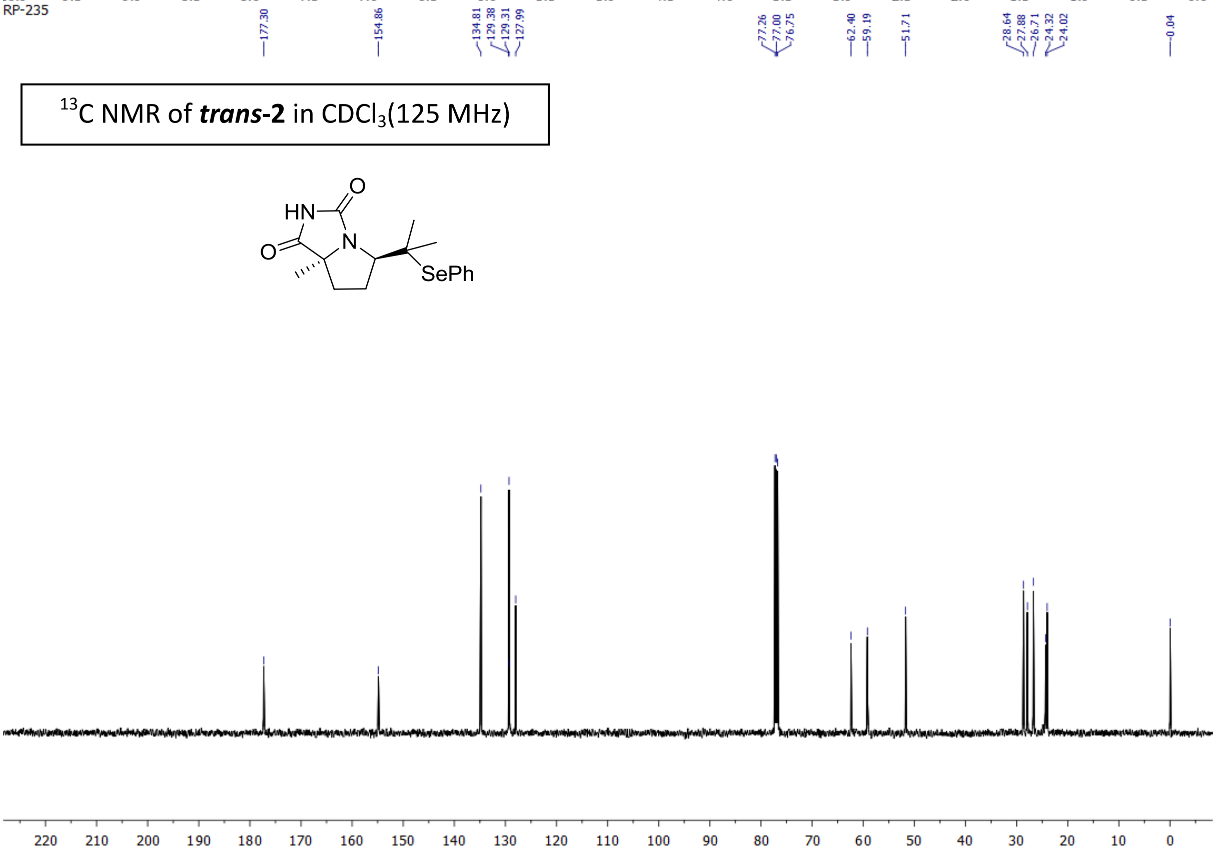

^{1}H-^{1}H COSY NMR of ***trans*-2** in $CDCl_3$(500 MHz)

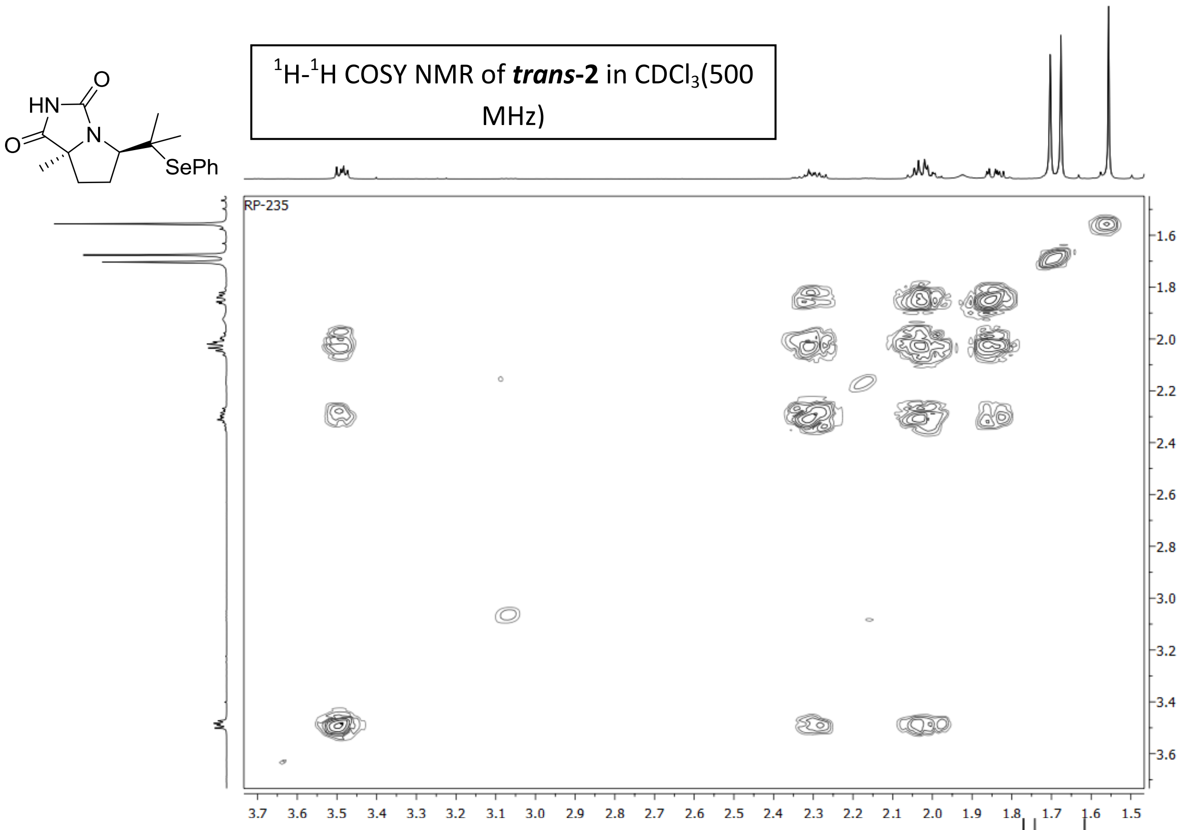

^{1}H-^{1}H NOESY NMR of ***trans*-2** in $CDCl_3$(500 MHz)

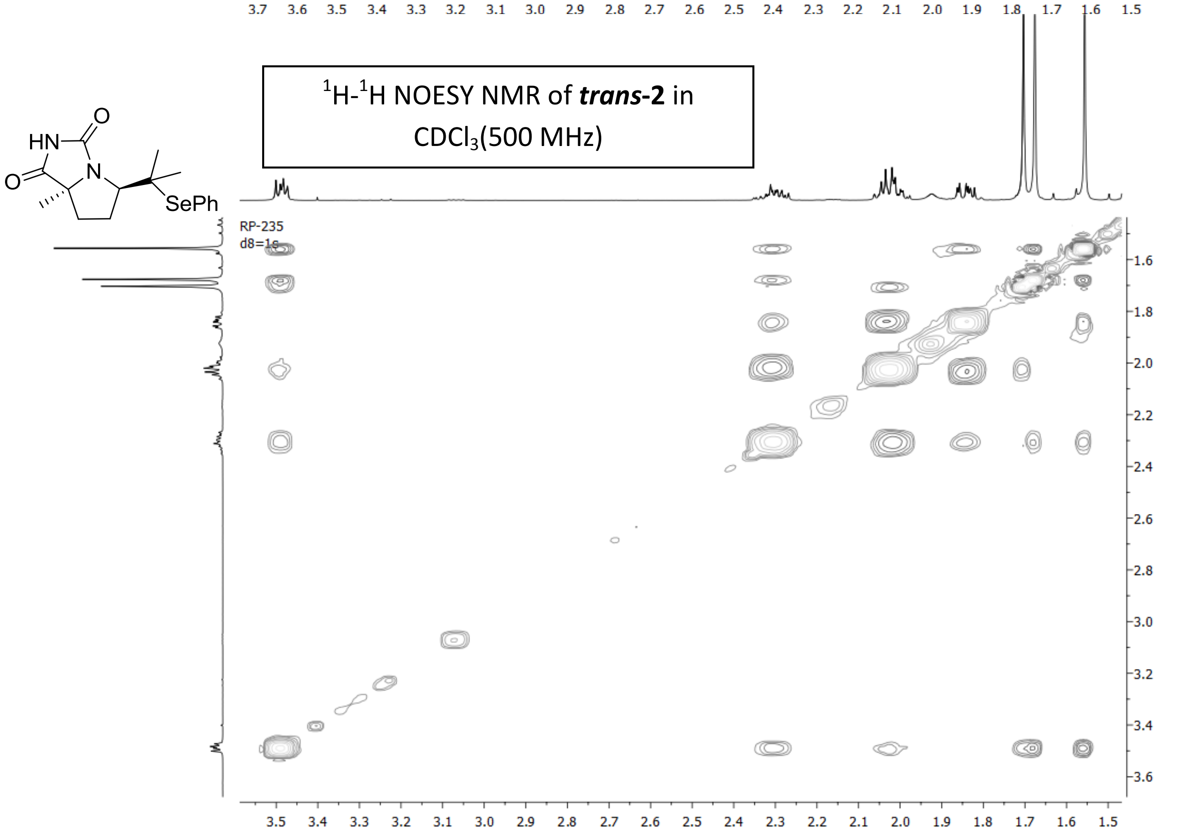

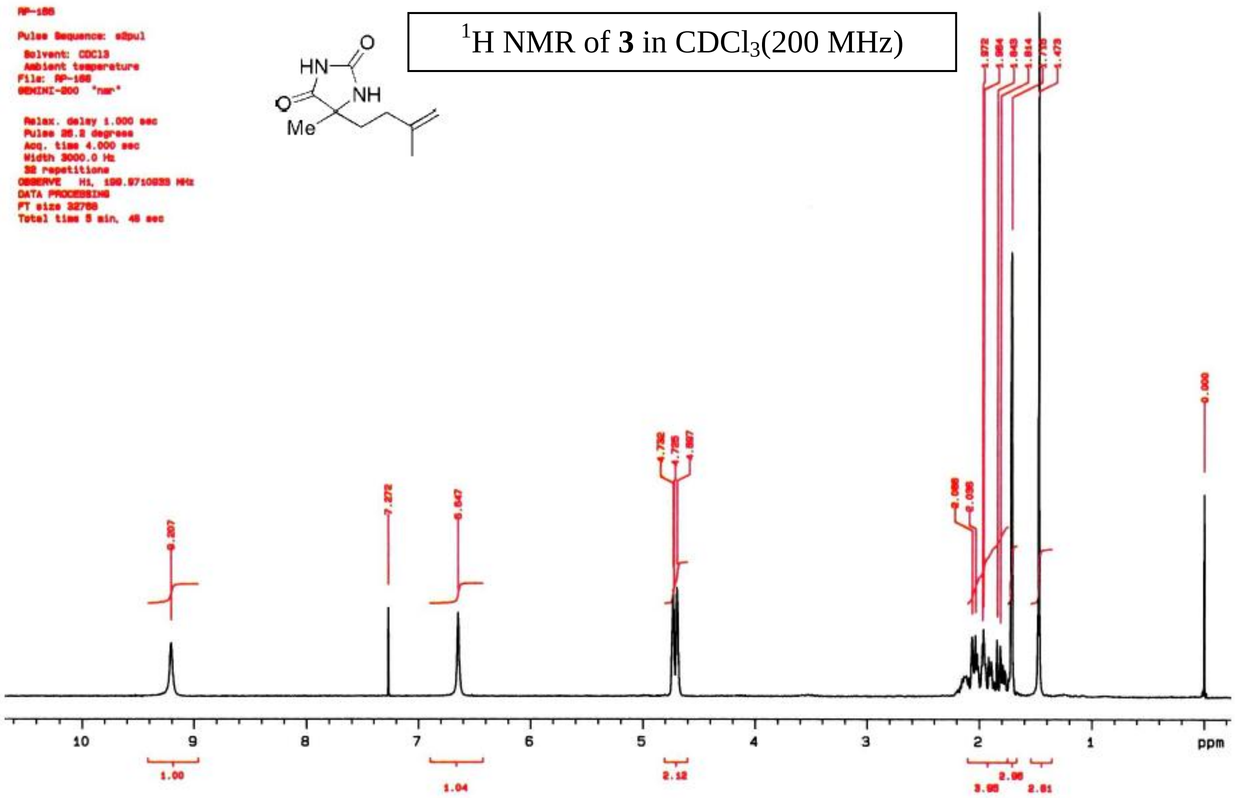

^{1}H NMR of **3** in $CDCl_3$(200 MHz)

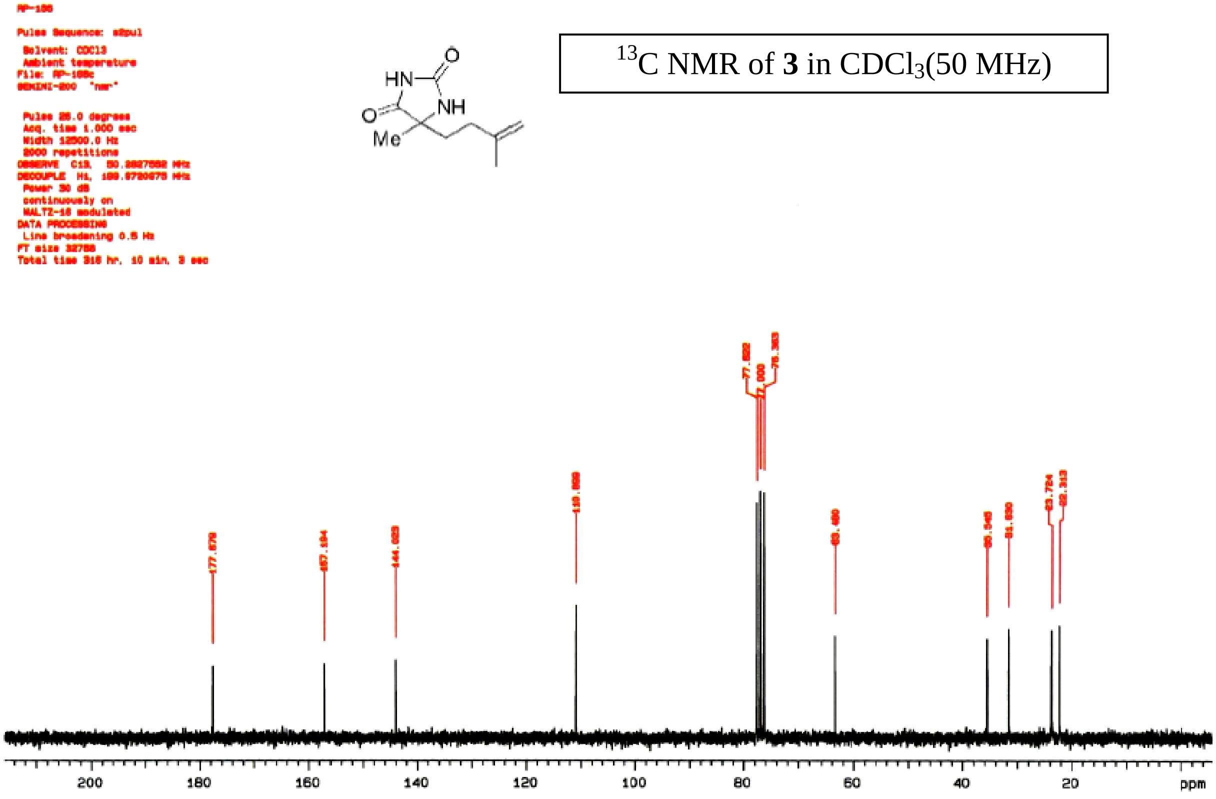

^{13}C NMR of **3** in $CDCl_3$(50 MHz)

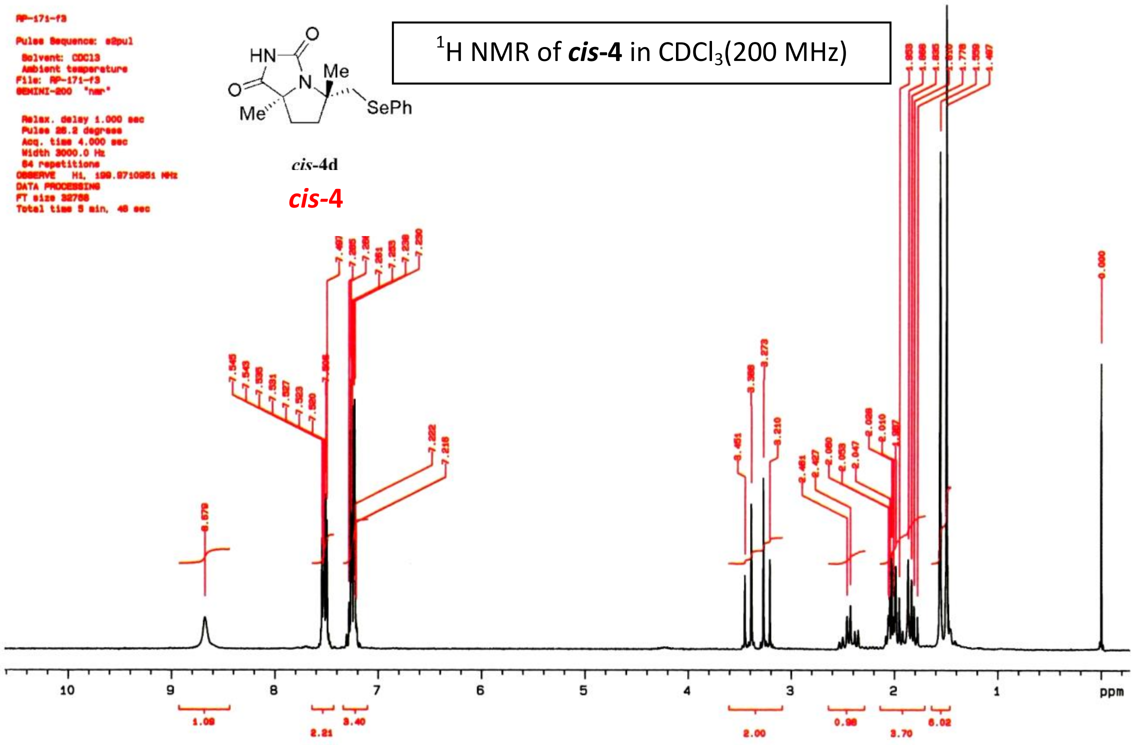

^1H NMR of ***cis*-4** in $CDCl_3$(200 MHz)

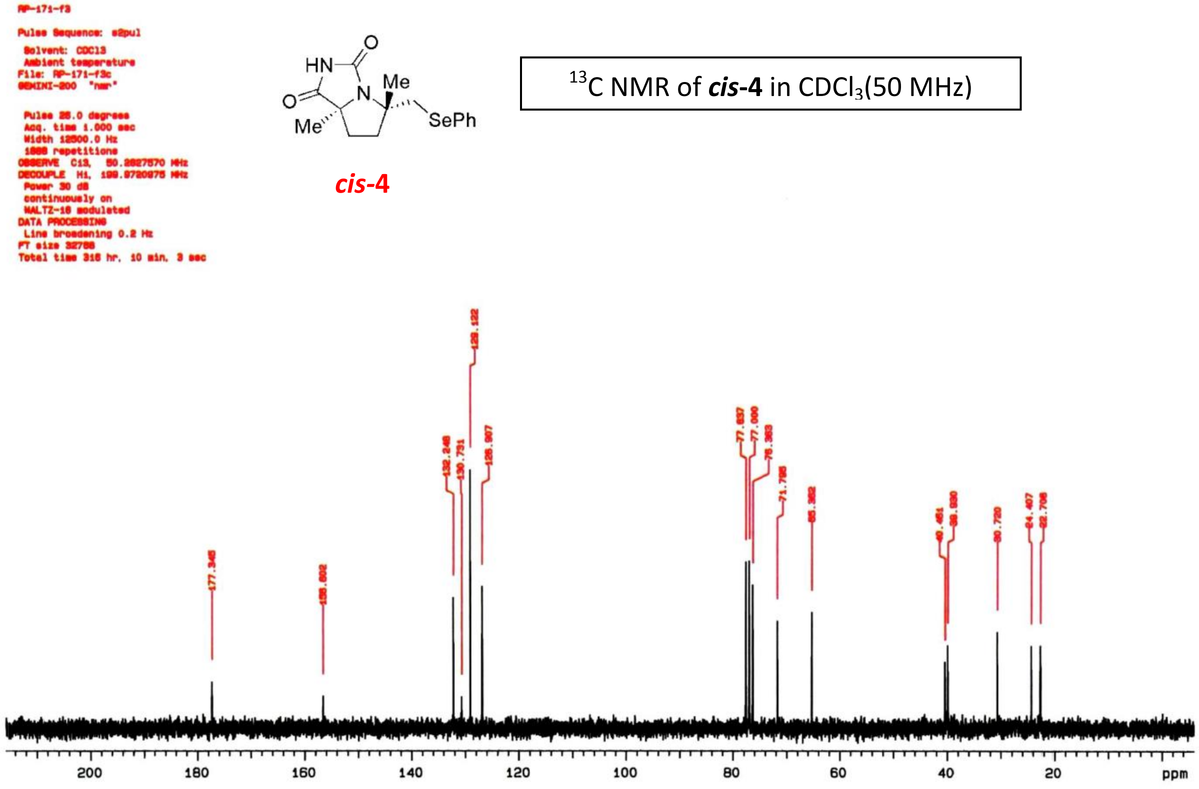

^{13}C NMR of ***cis*-4** in $CDCl_3$(50 MHz)

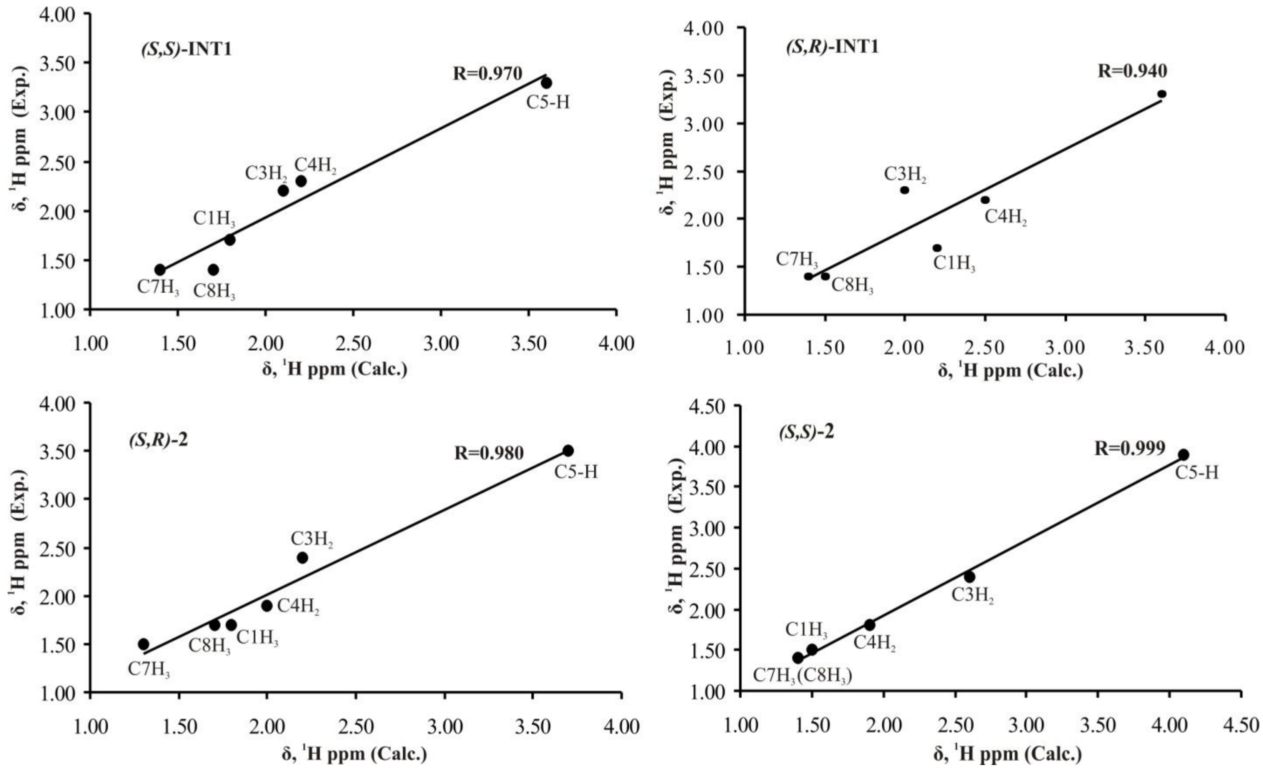

Figure S2. Correlation between calculated and experimental ^{1}H chemical shifts (ppm). See Table 1 for label assignments.

Table S1. Calculated proton chemical shifts for intermediates and products.

struct	***(S,R)*-INT1**	***(S,S)*-INT1**	***(S,R)*-INT1`**	***(S,S)*-INT1`**	***(S,R)*-INT2**	***(S,S)*-INT2**	***(S,R)*-INT3**	***(S,S)*-INT3**	***(S,R)*-INT3`**	***(S,S)*-INT3`**	***(S,R)*-2**	***(S,S)*-2**	***(S,R)*-2`**	***(S,S)*-2`**
bond														
C5-H	3.6	3.6	5.0	4.8	5.1	4.9	4.8	4.8	3.4	3.4	3.7	4.1	3.4	3.3
$C3H_2$	2.0	1.9	1.7	1.7	3.1	1.9	3.1	2.9	2.2	2.5	2.5	2.5	2.5	2.0
	2.9	2.4	2.7	3.1	3.1	2.5	2.4	3.0	1.9	2.2	2.0	2.7	1.9	1.4
$C4H_2$	2.3	2.3	1.5	2.0	0.7	2.2	2.6	2.5	1.7	2.3	2.2	1.9	1.5	1.
	1.8	2.1	0.9	2.0	1.7	2.4	2.4	2.3	2.1	2.2	1.8	1.9	1.8	1.8
$C1H_3$	2.7	1.9	2.0	1.6	2.4	2.5	1.7	2.1	2.0	2.6	2.6	1.8	1.3	2.4
	2.0	1.6	1.4	1.3	2.4	2.1	2.0	1.9	1.6	2.7	1.6	1.6	0.9	1.9
	2.0	1.8	1.3	1.4	1.5	2.0	1.6	1.5	2.1	1.5	1.3	1.1	1.9	1.3
$C7H_3$	1.6	1.6	1.3	1.7	1.6	1.6	2.4	1.4	2.2	2.2	1.4	1.4	2.3	1.0
	1.3	1.4	0.7	1.4	1.1	1.5	1.7	1.5	1.6	1.8	1.5	1.3	1.4	1.1
	1.2	1.2	0.8	1.2	1.0	1.2	1.7	1.7	1.5	1.6	1.0	1.6	1.1	1.8
$C8H_3$	1.2	1.7	1.7	1.5	2.5	2.3	1.5	1.8	2.5	1.4	2.1	1.5	2.3	1.8
	1.7	2.1	1.2	1.8	1.7	2.3	1.6	1.5	1.7	1.2	1.5	1.5	2.5	1.2
	1.6	1.2	1.4	0.8	2.3	0.7	1.5	1.2	1.8	1.4	1.4	1.2	1.6	1.6

Table S2. Calculated NBO charges for seleniranium cations *(S,R)*-INT2 and *(S,S)*-INT2.

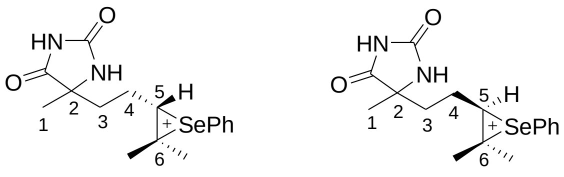

(S,R)-**INT2**		*(S,S)*-**INT2**	
Atom	Charge	Atom	Charge
C5	-0.214	C5	-0.243
C6	0.013	C6	0.026
Se	0.754	Se	0.774
N1	-0.660	N2	-0.660

Cartesian coordinates and electronic energies (in a. u.) of all the stationary points discussed in the text. All calculations have been performed at the SMD-(acetonitrile) B3LYP/6-311+G(d,p).

(S,R)-**INT1**

E=-3744.059208 au
Charge = 0; Multiplicity = 1

C	-4.13260000	0.30092700	-0.94295000
C	-3.31521600	0.09512000	1.21273300
C	-2.60866800	-0.96860400	0.34876600
O	-3.14984500	0.29285300	2.40024100
O	-4.81259700	0.73460100	-1.85866400
N	-3.21554200	-0.69439900	-0.95932100
H	-3.06008900	-1.25760600	-1.78315000
N	-4.16369100	0.77659700	0.38125500
C	-2.96948200	-2.37368000	0.85193700
H	-2.58975400	-3.13228300	0.16481700
H	-2.53105100	-2.54701400	1.83712900
H	-4.05374300	-2.48949100	0.92421200
C	-1.08738700	-0.67851100	0.36087700
H	-0.94532900	0.35071700	0.02789300
H	-0.76302700	-0.73167600	1.40039700

C	-0.27484600	-1.62558900	-0.52851700
H	-0.71180300	-1.62505000	-1.53163600
H	-0.36621300	-2.64966300	-0.15706400
C	1.22975100	-1.35192200	-0.72619700
H	1.53497100	-1.94574500	-1.59174000
C	2.21751100	-1.72969900	0.39975200
C	1.93893300	-1.15115000	1.77817700
H	1.93675900	-0.06042900	1.71568200
H	0.97991100	-1.47521500	2.17831300
H	2.72284700	-1.45105100	2.47658800
C	3.67887400	-1.56314300	-0.00084700
H	3.93595400	-0.50308900	-0.01965600
H	4.32568800	-2.05551600	0.72713400
H	3.87426900	-1.98175200	-0.99024900
H	-4.76177400	1.54004200	0.67369300
Se	1.59840600	0.44380700	-1.62046000
C	1.48747000	1.89609700	-0.32226300
C	2.60422400	2.25191900	0.44216200
C	0.33605400	2.68955200	-0.27115300
C	2.55502600	3.36987600	1.27580700
H	3.51282300	1.66603400	0.38632400
C	0.29414100	3.81142600	0.55788100
H	-0.52515000	2.43785400	-0.87832500
C	1.40024000	4.15007200	1.33726400
H	3.42398900	3.63370100	1.86892400
H	-0.60347500	4.41938000	0.59083100
H	1.36548600	5.02109500	1.98237900
Cl	2.01076100	-3.63434400	0.59802500

(S,S)-**INT1**

E=-3744.071900 au
Charge = 0; Multiplicity = 1

C	-4.22324000	-1.38865000	1.04619300
C	-3.83636900	-0.98910900	-1.20084800
C	-3.19606500	0.16899400	-0.40922300
O	-3.84321000	-1.12721100	-2.40808100
O	-4.64405600	-1.96293100	2.03729800
N	-3.51910100	-0.23630600	0.96366400
H	-3.31464600	0.32170600	1.78027700
N	-4.39318500	-1.83411700	-0.27808800
H	-4.89261800	-2.68527000	-0.50644800
C	-3.87411600	1.49571600	-0.77852400
H	-3.51874200	2.29672500	-0.12741800

H	-3.64578800	1.76246500	-1.81266100
H	-4.95836300	1.41841100	-0.66614000
C	-1.67154000	0.16514100	-0.66762600
H	-1.29092600	-0.83055900	-0.42096400
H	-1.51414600	0.31780600	-1.73943200
C	-0.89246700	1.22628200	0.11780300
H	-1.15543100	1.18867700	1.17993500
H	-1.17697000	2.21892200	-0.23670000
C	0.62983200	1.07242900	-0.04565600
H	0.87218300	0.70286300	-1.04135700
C	1.45028500	2.35417300	0.20855200
C	2.95048000	2.17243300	0.01861800
H	3.45426300	3.14022300	0.04617900
H	3.34845800	1.56256200	0.83291900
H	3.17979500	1.68088500	-0.92838100
C	1.12251500	3.07299400	1.50941300
H	1.38343700	2.41985900	2.34775200
H	1.70971300	3.98875800	1.59693000
H	0.06487400	3.32584600	1.58805500
Se	1.20683000	-0.41628600	1.19848200
C	2.56466000	-1.33806200	0.16078300
C	3.77008400	-1.66215000	0.79153500
C	2.33858900	-1.74643400	-1.15802400
C	4.74322300	-2.39113400	0.10538200
H	3.95225700	-1.34279500	1.81137000
C	3.32473400	-2.45392900	-1.84514800
H	1.40071600	-1.51769200	-1.65042600
C	4.52707300	-2.78181100	-1.21560000
H	5.67425000	-2.64019900	0.60307500
H	3.14602600	-2.75922700	-2.87059000
H	5.28816000	-3.33825800	-1.75127000
Cl	0.93407900	3.54557800	-1.20245000

(S,R)-**INT1`**

E= -3744.061904 au
Charge = 0; Multiplicity = 1

C	-4.73333600	0.08249300	-0.21920400
C	-3.25078200	-1.33929500	0.84593800
C	-2.50507400	-0.70232600	-0.34407400
O	-2.80275800	-2.14703100	1.63520900
O	-5.79366400	0.63394900	-0.46383500
N	-3.54893100	0.18738800	-0.86928400
H	-3.48510900	0.65371900	-1.76337000

N	-4.51623200	-0.81549300	0.84170100
C	-2.12453200	-1.78511400	-1.36351900
H	-1.69336200	-1.32899400	-2.25696500
H	-1.38907200	-2.46709000	-0.93274700
H	-3.00497000	-2.35945200	-1.66228600
C	-1.28762900	0.08359200	0.19802400
H	-1.65809600	0.79826900	0.93710200
H	-0.65235600	-0.63091300	0.72302500
C	-0.49261000	0.81502100	-0.89387400
H	-1.17991200	1.21148300	-1.64677800
H	0.15730800	0.11238400	-1.42011500
C	0.38755100	2.00006900	-0.48123500
H	0.74086400	2.48193000	-1.39039400
C	1.60097500	1.83620700	0.45796900
C	1.31052600	1.26741500	1.84179400
H	0.57652900	1.90209300	2.34886800
H	0.91863000	0.25277100	1.81663900
H	2.22108900	1.26680000	2.44431600
C	2.36959400	3.16192100	0.57420100
H	1.76171100	3.90273000	1.09881400
H	3.29082300	3.01838600	1.14147700
H	2.62535000	3.57029800	-0.40785500
H	-5.24107600	-1.06115600	1.50572100
Cl	-0.76574700	3.33088600	0.21764700
Se	2.96263900	0.67608800	-0.56424500
C	2.53732100	-1.18003500	-0.18025800
C	2.85001300	-1.74148500	1.06352300
C	2.04199400	-1.99527200	-1.20385200
C	2.64376200	-3.10305000	1.28558700
H	3.26044000	-1.12187100	1.85119100
C	1.85397400	-3.36035100	-0.98121700
H	1.80719100	-1.56806700	-2.17157700
C	2.14859100	-3.91507900	0.26402800
H	2.88385400	-3.53047600	2.25312700
H	1.47454600	-3.98656700	-1.78140300
H	1.99907100	-4.97521900	0.43662100

(S,S)-**INT1`**

E=-3744.069331 au
Charge = 0; Multiplicity = 1

C	5.29927200	-0.15545800	0.11467200
C	4.03007000	1.77922500	0.16583100
C	3.18604400	0.69167200	-0.52998000

O	3.70238900	2.93057700	0.37375400
O	6.26969400	-0.87628000	0.27956800
N	4.10552800	-0.45016400	-0.45211300
H	3.94713200	-1.33173000	-0.91904100
N	5.22098700	1.19432500	0.50406400
H	5.98355300	1.67164800	0.96993300
C	2.91410800	1.09627400	-1.98655100
H	2.44572700	0.27240600	-2.52855600
H	2.24612200	1.95956900	-2.02161600
H	3.84684400	1.35322700	-2.49472100
C	1.89968900	0.45351100	0.29330100
H	2.19674300	0.21303500	1.31795800
H	1.34680200	1.39675600	0.32848600
C	0.99305100	-0.65036000	-0.26306000
H	1.55725400	-1.57214900	-0.42568100
H	0.59715000	-0.34110800	-1.23245000
C	-0.20499500	-0.94401800	0.63933400
H	-0.58956700	-0.03545600	1.09797800
C	-1.37236500	-1.73148500	0.01572800
C	-2.44868900	-2.10472400	1.03354900
H	-3.33850900	-2.48154700	0.52551800
H	-2.08026800	-2.89473100	1.69328100
H	-2.73668800	-1.25433500	1.65511500
C	-0.92490200	-2.95485200	-0.78688500
H	-0.36430800	-3.63502400	-0.13737500
H	-1.79269800	-3.49512100	-1.17040500
H	-0.28428000	-2.69247100	-1.63181000
C	-3.31298700	0.70670400	-0.31272000
C	-4.68597500	0.46078000	-0.20091400
C	-2.75975900	1.84176000	0.29094200
C	-5.49665400	1.33925800	0.51951500
H	-5.12044400	-0.41207500	-0.67343400
C	-3.57366900	2.71197100	1.01716400
H	-1.70105400	2.04880400	0.19322100
C	-4.94181000	2.46278600	1.13245000
H	-6.56019600	1.14256000	0.60104900
H	-3.13776500	3.58766800	1.48583800
H	-5.57274200	3.14348100	1.69341200
Se	-2.21419700	-0.47998700	-1.38844700
Cl	0.45465000	-1.88150600	2.14329600

(S,R)-**INT2**

E=-3283.645055 au
Charge = 1; Multiplicity = 1

C	-3.66508400	0.52195400	-0.82057700
C	-3.16514400	-0.61570900	1.13102900
C	-2.37577000	-1.28781000	-0.01154800
O	-3.16008800	-0.93682200	2.30242100
O	-4.21591100	1.32903100	-1.54986400
N	-2.75088900	-0.42771900	-1.14020300
H	-2.57090000	-0.66841400	-2.10516000
N	-3.87081500	0.41320200	0.56627500
C	-2.87615900	-2.72709600	-0.20718400
H	-2.43047700	-3.17100800	-1.09958600
H	-2.60993800	-3.34006100	0.65662700
H	-3.96216700	-2.74226000	-0.32692300
C	-0.86516500	-1.19906600	0.31725800
H	-0.62674500	-0.15525400	0.52703600
H	-0.70527100	-1.76807100	1.23388000
C	0.02229800	-1.73908500	-0.80798100
H	-0.23509600	-1.26815900	-1.76158300
H	-0.18384100	-2.80802800	-0.94722100
C	1.53233100	-1.70244600	-0.69198300
H	1.98157000	-2.25877900	-1.51071100
C	2.40415400	-1.64189300	0.47746700
C	1.93435700	-1.31593000	1.86633800
H	2.77522800	-0.98767100	2.47899000
H	1.14569600	-0.57091500	1.90539800
H	1.54866400	-2.24508800	2.30362800
C	3.67943300	-2.44651000	0.41464000
H	4.45175400	-2.01113200	1.05012300
H	3.44746400	-3.44863900	0.79658800
H	4.05834900	-2.55403500	-0.60278800
H	-4.49092000	1.03207800	1.07540100
Se	2.65863000	0.06502900	-0.83580100
C	1.55513900	1.48830000	-0.13105700
C	0.51983500	1.98715800	-0.92545900
C	1.90283300	2.10048600	1.07559600
C	-0.19141900	3.10361600	-0.48905300
H	0.27412300	1.51377100	-1.86789500
C	1.17987000	3.21398300	1.50189500
H	2.72231700	1.71812900	1.67127400
C	0.13468100	3.71271900	0.72350000
H	-0.99766100	3.49643400	-1.09804400
H	1.43887100	3.69315800	2.43923700
H	-0.42165600	4.58087400	1.05868600

(S,S)-**INT2**

E=-3283.656778 au
Charge = 1; Multiplicity = 1

C	-4.41722000	-0.34788000	1.33492000
C	-3.82113400	-1.43037500	-0.62131600
C	-3.21586700	-0.01214700	-0.67487800
O	-3.72482600	-2.29233600	-1.47165200
O	-4.96281600	-0.17683300	2.41188200
N	-3.63743000	0.50933300	0.63067400
H	-3.57098300	1.48404000	0.88873800
N	-4.48480500	-1.52775100	0.57251000
H	-4.99272400	-2.34831400	0.88111100
C	-3.84243300	0.78086300	-1.83091700
H	-3.52840600	1.82578600	-1.78833500
H	-3.52825000	0.36248100	-2.78945100
H	-4.93309000	0.74951600	-1.77372800
C	-1.67913400	-0.13218100	-0.77935000
H	-1.32668100	-0.75180000	0.05116400
H	-1.44649800	-0.66387100	-1.70622700
C	-0.94132300	1.20992500	-0.75373400
H	-1.21653100	1.79015500	0.12925700
H	-1.21342000	1.81347300	-1.62841400
C	0.55586900	1.03978500	-0.83905100
H	0.89024300	0.31047800	-1.57189400
C	1.51934700	2.10067300	-0.56010800
C	2.79445600	2.14567600	-1.35127600
H	2.60204400	2.78650400	-2.22137000
H	3.60463800	2.59907500	-0.77798600
H	3.10017300	1.16656200	-1.71795000
C	1.08704500	3.40392000	0.05458400
H	1.93362800	3.90339500	0.52753100
H	0.72712100	4.04611700	-0.75912100
H	0.28164500	3.30000500	0.78096100
Se	1.47863400	0.53878700	0.94477100
C	2.86743600	-0.68127200	0.37045500
C	4.17038800	-0.43825300	0.81069600
C	2.55462300	-1.81898900	-0.37630600
C	5.17710400	-1.34612300	0.48220300
H	4.39843000	0.44464900	1.39572000
C	3.57197400	-2.71387800	-0.70325000
H	1.53831600	-2.00383600	-0.70196800
C	4.87979200	-2.47841400	-0.27595500
H	6.19201900	-1.16337900	0.81644600
H	3.33964500	-3.59654900	-1.28831200

H	5.66636100	-3.17896300	-0.53283000

(S,R)-**TS-INT3**

E=-3283.623817 au
Charge = 1; Multiplicity =1

C	3.39683000	-0.97993600	0.27065900
C	3.60505700	1.25997200	0.79241800
C	2.76780300	1.20661600	-0.48861500
O	3.97218400	2.24913900	1.37781700
O	3.63100000	-2.15840300	0.24000500
N	2.56249500	-0.27127400	-0.67636400
H	2.78597800	-0.58998600	-1.62111000
N	3.86645600	-0.04717100	1.16195500
C	3.52699900	1.81905700	-1.66753800
H	2.91977200	1.75687400	-2.57362500
H	3.73034400	2.87007900	-1.45684400
H	4.47512300	1.30360400	-1.83871800
C	1.37311700	1.82681700	-0.26976000
H	1.11603800	1.81433100	0.78992400
H	1.36324700	2.86463100	-0.60286300
C	0.37193400	0.98908700	-1.07175900
H	0.47954400	1.19323000	-2.13847800
H	-0.63920600	1.27008000	-0.77649600
C	0.57940200	-0.49253300	-0.84118400
H	0.69160100	-1.09944300	-1.73207700
C	-0.04112500	-1.25009700	0.28688200
Se	-1.90339000	-1.49332300	-0.62545000
C	-2.97689900	0.01375700	-0.05996100
C	-3.50647800	0.05178600	1.23555400
C	-3.32053500	1.00325800	-0.98844000
C	-4.35989900	1.09091300	1.60342900
H	-3.26141600	-0.72726200	1.94666400
C	-4.18227300	2.03483100	-0.61447000
H	-2.92313700	0.96539400	-1.99556900
C	-4.69803700	2.08222600	0.68069500
H	-4.76732700	1.11934100	2.60799500
H	-4.44932000	2.79864900	-1.33632000
H	-5.36695400	2.88549600	0.96907900
C	-0.13795800	-0.54816700	1.62865100
H	0.87188800	-0.42774500	2.03596000
H	-0.59999800	0.43652200	1.56156100
H	-0.71159000	-1.15552900	2.33037500
C	0.44095000	-2.69497100	0.41106000

H	1.43165400	-2.72425400	0.86426700
H	-0.23896400	-3.25843400	1.05191100
H	0.49835800	-3.19023800	-0.56168300
H	4.44817900	-0.30482100	1.95391300

(S,S)-**TS-INT3**

E=-3283.631713 au
Charge = 1; Multiplicity =1

C	2.31604200	1.39399900	1.22879000
C	3.87027100	1.28736900	-0.47839800
C	3.26955400	-0.11725600	-0.36262200
O	4.74424800	1.64578300	-1.23141800
O	1.70814100	1.81548400	2.17873900
N	2.25760100	0.05522500	0.72428700
H	2.32069100	-0.62598600	1.48036500
N	3.19753400	2.08559500	0.42597800
H	3.39126200	3.07297800	0.56264400
C	4.32306000	-1.16396600	-0.00329600
H	3.86374300	-2.15298700	0.06016700
H	5.08946200	-1.18693100	-0.77974000
H	4.79830100	-0.93247400	0.95300800
C	2.48428500	-0.45112500	-1.65599300
H	2.13402000	0.46964900	-2.12986900
H	3.12737500	-0.96932500	-2.36756400
C	1.28184700	-1.31073600	-1.27027900
H	1.59457000	-2.29900500	-0.93209800
H	0.65019100	-1.44628500	-2.15116700
C	0.47709600	-0.60635200	-0.21438800
H	0.18048800	0.41298700	-0.44224300
C	-0.33622400	-1.27480700	0.82121700
Se	-1.95335500	-1.52858900	-0.47425100
C	-2.90337900	0.15300800	-0.35025500
C	-3.88602400	0.32156000	0.63138300
C	-2.67104800	1.15847500	-1.29494000
C	-4.62510400	1.50381500	0.67269500
H	-4.07464100	-0.46234300	1.35462800
C	-3.41319500	2.33816000	-1.24490400
H	-1.92033700	1.02098400	-2.06366400
C	-4.38866100	2.51206000	-0.26214600
H	-5.38617500	1.63343800	1.43416100
H	-3.23120800	3.11774800	-1.97633200
H	-4.96576900	3.42943200	-0.22793700
C	-0.73308000	-0.41116600	2.00551400
H	-1.55817700	-0.87565400	2.54804600

H	0.11786900	-0.33145400	2.68808300
H	-1.02417400	0.59603300	1.70799700
C	0.08613400	-2.68037000	1.23131100
H	1.06074300	-2.63572400	1.72993700
H	-0.63671700	-3.09027800	1.93818000
H	0.16801900	-3.36465900	0.38538200

(S,R)-**TS-INT3`**

E=-3283.626234 au
Charge = 1; Multiplicity =1

C	-2.75682600	1.65371400	-0.81495900
C	-3.83166800	0.79842100	1.03886000
C	-3.07133600	-0.38310800	0.42500200
O	-4.56504900	0.76968800	1.99748700
O	-2.49223000	2.40603600	-1.71449900
N	-2.37484100	0.25461700	-0.75827800
H	-2.68870700	-0.17675000	-1.62783700
N	-3.49505300	1.92283700	0.31073100
H	-3.88440700	2.84429300	0.48723200
C	-4.03045800	-1.47514500	-0.05329700
H	-3.46774900	-2.32011600	-0.45549200
H	-4.62727100	-1.82634800	0.78974300
H	-4.70468300	-1.09718200	-0.82618700
C	-2.07784700	-0.89217700	1.49433300
H	-1.70917600	-0.04823900	2.08537700
H	-2.64353800	-1.53417700	2.17222800
C	-0.87272900	-1.65013200	0.94073900
H	-1.17895800	-2.48382400	0.30471400
H	-0.32889100	-2.07474500	1.78614900
C	0.06243000	-0.69218900	0.20448900
H	0.28680000	0.16520600	0.83693100
C	-0.37822400	-0.22315500	-1.14834600
Se	1.86969100	-1.58712800	-0.05933800
C	3.06265500	-0.09439600	0.26339200
C	3.94054300	0.29904900	-0.75141600
C	3.10212300	0.53819100	1.51090300
C	4.85559400	1.32727100	-0.51670700
H	3.91023100	-0.19085800	-1.71746200
C	4.00579300	1.57649400	1.73090000
H	2.43536600	0.22255100	2.30489200
C	4.88500400	1.97060800	0.71986800
H	5.53640800	1.62851700	-1.30526100
H	4.03041800	2.06948100	2.69656300

H	5.59103400	2.77413300	0.89776400
C	0.17866600	1.08128600	-1.61347300
H	1.22556100	0.88363000	-1.87691800
H	-0.32236900	1.44742400	-2.50669900
H	0.17561400	1.83898300	-0.83017400
C	-0.66504600	-1.22993900	-2.22717100
H	-1.23946000	-0.78262600	-3.03968200
H	0.29782400	-1.53075200	-2.64985200
H	-1.16810600	-2.12298800	-1.85830800

(S,S)-**TS-INT3`**

E=-3283.626232 au
Charge = 1; Multiplicity =1

C	-4.03056900	-1.47528800	0.05356900
H	-4.70487600	-1.09711800	0.82628400
H	-4.62729600	-1.82673400	-0.78943600
H	-3.46788000	-2.32013800	0.45605500
C	-3.07139000	-0.38337800	-0.42496800
C	-2.07784100	-0.89279700	-1.49407400
C	-0.87268400	-1.65044800	-0.94014900
H	-1.70922000	-0.04905700	-2.08543200
H	-2.64345800	-1.53510400	-2.17173700
C	-0.37812100	-0.22240000	1.14822300
C	0.06247300	-0.69213900	-0.20437900
H	-0.32885400	-2.07544900	-1.78536800
H	-1.17886300	-2.48383300	-0.30368900
C	-0.66460100	-1.22855300	2.22772700
H	0.29834400	-1.52858400	2.65078100
H	-1.23934700	-0.78091300	3.03982400
H	-1.16720500	-2.12209100	1.85941200
C	0.17856000	1.08246200	1.61240300
H	-0.32217500	1.44895300	2.50565100
H	1.22564500	0.88528500	1.87546000
H	0.17490400	1.83971200	0.82867000
H	0.28690000	0.16492000	-0.83725300
Se	1.86969600	-1.58704400	0.05994000
C	3.06273700	-0.09449900	-0.26332400
C	3.94052400	0.29930500	0.75143600
C	3.10242300	0.53756500	-1.51109300
C	4.85568000	1.32736400	0.51643100
H	3.91004600	-0.19020800	1.71767700
C	4.00620400	1.57570900	-1.73139500
H	2.43575500	0.22165100	-2.30504700

C	4.88530500	1.97018200	-0.72040800
H	5.53640700	1.62888500	1.30495500
H	4.03099900	2.06829500	-2.69725800
H	5.59141700	2.77358400	-0.89854200
C	-3.83169000	0.79797500	-1.03920800
N	-3.49529800	1.92256300	-0.31123700
H	-3.88486100	2.84391100	-0.48783500
N	-2.37498100	0.25469200	0.75817600
H	-2.68858200	-0.17665900	1.62783400
C	-2.75731500	1.65367000	0.81467000
O	-2.49309200	2.40614300	1.71419700
O	-4.56486800	0.76898800	-1.99798600

(S,R)-**INT3**

E=-3283.635410 au
Charge = 1; Multiplicity = 1

C	-2.82232300	-0.73893000	1.24473200
C	-4.10709800	0.63960800	-0.10973800
C	-2.72658100	1.29573200	-0.13545600
O	-5.09834200	1.03709900	-0.66301100
O	-2.50937500	-1.56263200	2.04886100
N	-1.87391300	0.30781000	0.69963800
N	-4.03076500	-0.50012700	0.67749100
C	-2.76888000	2.67885800	0.50543900
H	-1.75769800	3.08651000	0.56403900
H	-3.37125300	3.33611900	-0.12430900
H	-3.20862000	2.64881300	1.50481700
C	-2.11981600	1.25988000	-1.55396700
H	-2.90550400	1.32441200	-2.30648600
H	-1.45950400	2.12077400	-1.67382400
C	-1.31983000	-0.04014700	-1.61765800
H	-0.57558900	-0.02557000	-2.41122200
H	-1.97138200	-0.90446400	-1.77043200
C	-0.64734900	-0.09925100	-0.25281100
H	-0.03066900	0.79010200	-0.11887500
C	0.19835700	-1.31831100	0.15466700
Se	1.86474800	-1.19772100	-1.05466900
C	2.94392500	0.17030000	-0.19912300
C	2.77804600	1.51855900	-0.53634800
C	3.94500800	-0.20267800	0.70482600
C	3.59811600	2.48757500	0.04333900
H	2.01754000	1.81053200	-1.25063900
C	4.76545100	0.77021300	1.27688300

H	4.08349800	-1.24643100	0.96002700
C	4.59116800	2.11528200	0.94995600
H	3.46409500	3.53118000	-0.21991600
H	5.54041900	0.47483300	1.97578700
H	5.23033200	2.86962500	1.39541500
C	-0.39380200	-2.67861600	-0.22450000
H	-1.35739900	-2.84245700	0.26217600
H	-0.54436900	-2.77595700	-1.30231500
H	0.27774700	-3.47636700	0.09875000
C	0.64992500	-1.25799700	1.61297100
H	-0.19169100	-1.45629200	2.27997000
H	1.41143800	-2.01597300	1.80508400
H	1.06448500	-0.28041800	1.86828400
H	-4.84090200	-1.07438300	0.89990800
H	-1.46782000	0.77792100	1.51653900

(S,S)-**INT3**

E=-3283.641007 au
Charge = 1; Multiplicity = 1

C	2.04748200	1.57215200	0.97556200
C	3.99832600	1.15350600	-0.21671900
C	3.19810000	-0.13714700	-0.35531100
O	5.08154000	1.38576700	-0.68458000
O	1.17132300	2.11246100	1.57486400
N	2.00003700	0.10929600	0.57323100
H	2.12332100	-0.42666400	1.44124800
N	3.23307500	2.05037400	0.52275000
H	3.51077900	3.01577200	0.68627500
C	3.99687200	-1.36329700	0.06390800
H	3.40443500	-2.26991100	-0.06542400
H	4.88224200	-1.43335400	-0.56976500
H	4.31721300	-1.28787800	1.10597300
C	2.53512500	-0.24386800	-1.74475700
H	2.37116500	0.75235000	-2.16363000
H	3.17308000	-0.80393500	-2.42728100
C	1.19852300	-0.93511100	-1.47045000
H	1.34026900	-2.01111700	-1.37183800
H	0.48825100	-0.76141700	-2.27595800
C	0.66949900	-0.33089300	-0.16069700
H	0.15754400	0.61008700	-0.35579600
C	-0.22638000	-1.21190600	0.73995000
Se	-1.90809900	-1.60889600	-0.35635900
C	-2.87072900	0.07734200	-0.30913500

C	-3.85064400	0.28665500	0.66746800
C	-2.64758100	1.05012500	-1.29016000
C	-4.59323700	1.46802600	0.66852700
H	-4.03492400	-0.46886300	1.42169000
C	-3.39093300	2.23117800	-1.28175400
H	-1.90366000	0.88675400	-2.06065400
C	-4.36344500	2.44176900	-0.30363100
H	-5.35172200	1.62353400	1.42797900
H	-3.21327100	2.98197100	-2.04398900
H	-4.94276900	3.35845100	-0.30230100
C	-0.62408200	-0.50661700	2.03585100
H	-1.33659500	-1.12024000	2.58997500
H	0.25082100	-0.36557100	2.68033700
H	-1.06909400	0.47160600	1.85418700
C	0.35071500	-2.60023300	1.03960300
H	1.31424400	-2.51782900	1.55414200
H	-0.32554500	-3.14095700	1.70449100
H	0.49922000	-3.20394400	0.14288100

(S,R)-**INT3`**

E=-3283.630274 au
Charge = 1; Multiplicity = 1

C	2.82225800	0.12199900	1.57432700
C	3.56786900	1.61054500	-0.04379800
C	2.62686600	0.68036900	-0.81271500
O	4.19448700	2.53366700	-0.49425500
O	2.60228400	-0.37712800	2.63068100
N	2.42788500	-0.47496100	0.20946200
N	3.51685900	1.25272700	1.29210500
H	3.97109000	1.78522000	2.03083400
C	3.26558900	0.21814700	-2.11671900
H	2.54132700	-0.24759200	-2.78126200
H	3.66339300	1.10203100	-2.61836600
H	4.09227400	-0.47272500	-1.93553800
C	1.30421900	1.45817500	-1.00094500
H	1.10765700	2.06741700	-0.11369500
H	1.45790400	2.14222900	-1.83757100
C	0.09783400	0.55344700	-1.25029100
H	0.19527000	0.01559500	-2.19542000
H	-0.78571400	1.18881100	-1.33239400
C	-0.07863300	-0.41261800	-0.08387700
H	-0.20048600	0.14711700	0.84541000

C	1.10545100	-1.38451600	0.09104500
Se	-1.80241500	-1.44847000	-0.25599100
C	-3.02049000	0.03476700	0.04333600
C	-3.11555200	0.64173500	1.30006500
C	-3.85064600	0.45406500	-1.00023300
C	-4.02701800	1.67786900	1.50243000
H	-2.48585600	0.30795400	2.11662100
C	-4.77333900	1.48004900	-0.78552800
H	-3.77636700	-0.01341400	-1.97499900
C	-4.85893300	2.09644500	0.46219800
H	-4.09415700	2.14961600	2.47674900
H	-5.41669200	1.80029300	-1.59783400
H	-5.57135200	2.89753600	0.62520900
C	0.98737300	-2.25775400	1.34353400
H	0.25973900	-3.04047600	1.12614400
H	1.93547800	-2.74747300	1.57296300
H	0.64680900	-1.70545800	2.21573000
C	1.34552600	-2.30500100	-1.11401900
H	2.26442400	-2.88143600	-0.97806800
H	0.51962500	-3.01704000	-1.16197300
H	1.39045600	-1.78669100	-2.06773400
H	3.18966200	-1.14655600	0.03366200

(S,S)-**INT3`**

E= -3283.630990 au
Charge = 1; Multiplicity = 1

C	3.18259500	-1.05067600	1.02529300
C	3.76175600	1.15140800	0.57689300
C	2.64484900	0.86846900	-0.42822400
O	4.38393400	2.17559100	0.68375700
O	3.18625800	-2.15625800	1.47273700
N	2.28650500	-0.60818000	-0.11899800
H	2.59565800	-1.16227200	-0.92761700
N	3.93285200	0.02516600	1.36766800
C	3.17856800	0.99256900	-1.85752800
H	2.38748900	0.81950200	-2.58782600
H	3.55963500	2.00537500	-1.99558700
H	3.99281500	0.28617900	-2.03835200
C	1.46557500	1.80330400	-0.12090400
H	1.34378300	1.87589400	0.96066300
H	1.75368700	2.79700500	-0.46710200
C	0.13601700	1.37636600	-0.77443200
H	0.00376300	1.87793300	-1.73424900

H	-0.66758500	1.71976800	-0.12223100
C	0.02992000	-0.13260300	-1.01522500
H	0.49271800	-0.38236700	-1.97321500
C	0.72574100	-1.01425600	0.04795500
Se	-1.84774700	-0.68836900	-1.48652100
C	-2.97459700	0.06127700	-0.08930700
C	-3.46355800	-0.77358800	0.92188500
C	-3.39058600	1.39608200	-0.15014400
C	-4.34714300	-0.26929000	1.87668000
H	-3.15943100	-1.81255200	0.96360300
C	-4.26667400	1.89741800	0.81390300
H	-3.04060600	2.04000500	-0.94780700
C	-4.74455400	1.06705700	1.82787200
H	-4.72340400	-0.92235500	2.65661600
H	-4.58086900	2.93428200	0.76486500
H	-5.42974800	1.45740100	2.57230700
C	0.27274600	-0.77061300	1.48281000
H	0.77409600	-1.44501000	2.17803400
H	0.41584700	0.25415400	1.82061900
H	-0.79406300	-0.99115800	1.52798500
C	0.66661300	-2.49993900	-0.30756700
H	1.30311000	-3.09071700	0.34887700
H	-0.35968300	-2.84384100	-0.18766600
H	0.96271800	-2.67076000	-1.34657500
H	4.63281700	-0.03337300	2.10396700

(S,R)-**TS-2**

E=-3744.053150 au
Charge = 0; Multiplicity = 1

C	-2.75133500	-0.58369200	0.98620300
C	-3.95050500	0.38635000	-0.73497900
C	-2.49534700	0.79751700	-0.95113000
O	-4.90227800	0.68641100	-1.41934200
O	-2.54337800	-1.23860200	1.97720500
N	-1.77913800	0.18332600	0.25418900
H	-1.38574500	1.23299800	1.20999100
N	-3.98524200	-0.38063400	0.40574900
C	-2.39266300	2.32112300	-1.01474900
H	-1.34570000	2.62499400	-1.05986400
H	-2.88625900	2.65816200	-1.92907100
H	-2.87097500	2.80560300	-0.16256300
C	-1.87593400	0.11522500	-2.20174500
H	-2.64848600	-0.19874300	-2.90447700

H	-1.23450500	0.83839400	-2.70953000
C	-1.04574800	-1.04469000	-1.65576800
H	-0.23927900	-1.32913400	-2.32946300
H	-1.67110000	-1.92387800	-1.48038300
C	-0.51263000	-0.50128500	-0.32650400
H	0.11842600	0.36641000	-0.52995500
C	0.32236700	-1.46199000	0.55800200
Se	2.10691300	-1.71465700	-0.45187200
C	2.95349000	0.02866700	-0.33992200
C	2.80587500	0.94926200	-1.38437000
C	3.77602200	0.34313200	0.74851600
C	3.46417100	2.17876200	-1.33113700
H	2.18481900	0.70703100	-2.23850600
C	4.43131400	1.57393000	0.79688000
H	3.90733800	-0.37045400	1.55293000
C	4.27512200	2.49371300	-0.24074700
H	3.34461200	2.88667700	-2.14409200
H	5.06586100	1.81050400	1.64410900
H	4.78785800	3.44850000	-0.20254200
C	-0.19843300	-2.90169900	0.63786500
H	-1.17268500	-2.92760500	1.12803500
H	-0.29769200	-3.36691200	-0.34515300
H	0.48738800	-3.50991800	1.23247900
C	0.63766100	-0.90388400	1.94316900
H	-0.27564700	-0.84949100	2.53878400
H	1.33991200	-1.56155700	2.45990400
H	1.07090800	0.09559900	1.89478800
H	-4.83385900	-0.77140100	0.80399500
Cl	-1.04852200	2.27580000	2.17142600

(S,S)-**TS-2**

E=-3744.057218 au
Charge = 0; Multiplicity = 1

C	1.81806200	1.32411700	1.10724300
C	3.58960100	1.61876500	-0.34752900
C	2.74921400	0.48252000	-0.92036100
O	4.59770300	2.09487400	-0.81485200
O	1.02101900	1.56068700	1.97702800
N	1.75931700	0.19478800	0.20284100
H	2.32887500	-0.79631500	1.05533100
N	2.96045300	2.03223000	0.81018600
H	3.25597500	2.83288400	1.36038800
C	3.60736100	-0.71387100	-1.32129000

H	2.98395800	-1.53230800	-1.68357000
H	4.27172700	-0.40382900	-2.13008700
H	4.21724000	-1.07422200	-0.49320700
C	1.83529800	0.97470100	-2.06339400
H	1.57490900	2.02607300	-1.91030700
H	2.32784000	0.87807400	-3.03079300
C	0.59329600	0.09413800	-1.92406500
H	0.76531000	-0.88213900	-2.37816300
H	-0.27314400	0.53923900	-2.40941000
C	0.35168300	-0.03869400	-0.40807700
H	-0.24550200	0.80433600	-0.06156100
C	-0.35388400	-1.32750700	0.07869000
Se	-2.17110900	-1.41762800	-0.88200900
C	-3.13102300	0.11283400	-0.16634600
C	-3.94870100	-0.03670800	0.95962800
C	-3.06985400	1.34956100	-0.81892200
C	-4.68972500	1.04677800	1.43377000
H	-4.00957600	-0.99449300	1.46234300
C	-3.81165000	2.43011800	-0.33964200
H	-2.45047800	1.46891200	-1.69981300
C	-4.62123300	2.28094500	0.78683500
H	-5.32093300	0.92328800	2.30710200
H	-3.75890800	3.38541800	-0.85060600
H	-5.19975700	3.12080500	1.15563400
C	-0.61560100	-1.30975600	1.58371500
H	-1.19173900	-2.18963200	1.87769100
H	0.32593100	-1.33028200	2.13795500
H	-1.16199400	-0.41684900	1.89011600
C	0.32609200	-2.62939800	-0.35581300
H	1.34074000	-2.68201800	0.04604700
H	-0.22540500	-3.48800000	0.03440900
H	0.38759300	-2.73264900	-1.44078900
Cl	3.06548400	-1.64951800	2.03849200

(S,R)-**TS-2`**

E=-3744.044500 au
Charge = 0; Multiplicity = 1

C	3.32782300	-0.42336300	0.73590300
C	3.40911900	1.68481000	-0.20189600
C	1.94954700	1.24186200	-0.31547900
O	3.87906000	2.73479000	-0.57661200
O	3.70705600	-1.42143500	1.29336100
N	1.98575300	-0.18697100	0.24656300

H	2.00166900	-1.09689800	-0.84154300
N	4.10167300	0.66297900	0.40277500
H	5.09805900	0.68946900	0.59719200
C	1.53978000	1.29527800	-1.79404800
H	0.53300600	0.90475100	-1.93758000
H	1.55632500	2.33967500	-2.11049300
H	2.22825500	0.73284700	-2.42452100
C	1.07855900	2.16208900	0.55387700
H	1.56036200	2.30068700	1.52235300
H	1.05898100	3.14221400	0.07447600
C	-0.35540600	1.63800500	0.72759800
H	-0.97043500	1.97788500	-0.10565600
H	-0.77695800	2.09562300	1.62627000
C	-0.46617900	0.09953100	0.85135900
H	-1.15291900	-0.14962200	1.65693800
C	0.85417300	-0.61704300	1.23428300
Se	-1.44211300	-0.71828500	-0.73727000
C	-3.22389400	-0.11467600	-0.24881200
C	-3.87028800	-0.63539900	0.87802900
C	-3.88590000	0.80700500	-1.06549600
C	-5.16392000	-0.22094100	1.19284700
H	-3.36849700	-1.36332000	1.50527000
C	-5.18804200	1.20574200	-0.75445400
H	-3.38877100	1.21377500	-1.93837900
C	-5.82658400	0.69752100	0.37596600
H	-5.65751000	-0.62539600	2.06998700
H	-5.69664400	1.91947500	-1.39355700
H	-6.83544900	1.01204600	0.61936600
C	1.19800000	-0.21113700	2.68707400
H	0.37685000	-0.53652100	3.32921400
H	2.10077100	-0.71709600	3.02917400
H	1.32670000	0.86005700	2.82702300
C	0.70348400	-2.14426500	1.21084600
H	1.59647900	-2.62452000	1.60875100
H	-0.14439000	-2.40981400	1.84656100
H	0.51175400	-2.53283800	0.21352300
Cl	2.19362700	-2.07881100	-1.96424200

(S,S)-**TS-2`**

E=-3744.048649 au
Charge = 0; Multiplicity = 1

C	1.83237100	2.11872300	1.24928400
H	2.38826700	1.66732600	2.07079600

H	2.22566200	3.12404800	1.08428800
H	0.78702500	2.21628700	1.53444800
C	1.99871200	1.34404000	-0.05697500
C	0.97953400	1.78469000	-1.14092800
C	-0.40002900	1.14618600	-0.96432600
H	1.36306900	1.51717500	-2.13054200
H	0.90668400	2.87405600	-1.10455000
C	0.61268200	-0.89787300	0.21976900
C	-0.28252900	-0.37524400	-0.93557400
H	-1.02525700	1.44975900	-1.80611900
H	-0.88240700	1.51850600	-0.05863200
C	0.05193300	-0.56385000	1.61100700
H	-0.86161000	-1.14172900	1.74810300
H	0.75232400	-0.86418500	2.39154500
H	-0.19568500	0.48467300	1.75113800
C	0.80088500	-2.42319500	0.15953300
H	1.61614600	-2.74475200	0.80966100
H	-0.11675800	-2.88490000	0.52470000
H	0.99332500	-2.78623000	-0.84701200
H	0.15801700	-0.71698100	-1.87628800
Se	-2.08138300	-1.28519000	-1.06510000
C	-3.28808900	-0.11488000	-0.08354200
C	-3.88196800	0.97692900	-0.72658800
C	-3.65232200	-0.42667800	1.23053400
C	-4.81480300	1.76369700	-0.04939700
H	-3.62268600	1.21047900	-1.75215800
C	-4.58915300	0.36041700	1.90177900
H	-3.21092400	-1.28122600	1.72856100
C	-5.16872800	1.45800900	1.26498800
H	-5.26874700	2.61005900	-0.55340400
H	-4.86634100	0.11183100	2.92054000
H	-5.89743600	2.06775200	1.78780700
C	3.38395800	1.59002300	-0.66323600
N	3.80472500	0.38653000	-1.17207300
H	4.64358300	0.26488500	-1.73076900
N	1.99184600	-0.18554900	0.07265900
H	2.73264400	-0.51267800	1.22482800
C	2.95714400	-0.66778000	-0.90114100
O	3.08030500	-1.78437700	-1.33002500
O	3.97108500	2.64429700	-0.74383200
Cl	3.64255400	-0.89489500	2.35835800

***(S,R)*-2**

E=-3283.219900 au

Charge = 0; Multiplicity = 1

C	2.81905100	-0.72337800	-1.07060400
C	4.04581300	0.96468100	-0.06480700
C	2.56697600	1.21794100	0.23013600
O	4.99634000	1.62399300	0.30889900
O	2.65945000	-1.77475700	-1.66629900
N	1.88785700	0.11752000	-0.50998800
N	4.08135400	-0.13635800	-0.87456400
C	2.17026200	2.59796100	-0.30465000
H	1.09430900	2.74670500	-0.19061500
H	2.68605700	3.37640800	0.26281400
H	2.43153200	2.70173800	-1.36090100
C	2.15916500	1.00592600	1.72287300
H	3.03140900	0.86417100	2.36263400
H	1.62939100	1.89270200	2.07583900
C	1.23096500	-0.22559500	1.71622400
H	0.45063900	-0.15670900	2.47341300
H	1.80399000	-1.13855800	1.89586700
C	0.66250400	-0.24307700	0.28535900
H	0.02279300	0.63497600	0.16533100
C	-0.19012700	-1.44658300	-0.17294800
Se	-1.95643700	-1.33761400	0.88712900
C	-2.84250500	0.21658900	0.13147000
C	-2.68514300	1.47490700	0.72442000
C	-3.69951800	0.07198800	-0.96612500
C	-3.36682600	2.58002800	0.21218700
H	-2.03730200	1.59222600	1.58479800
C	-4.37840900	1.17996700	-1.47457400
H	-3.83882800	-0.90131900	-1.42119900
C	-4.21195300	2.43507500	-0.88812000
H	-3.23888700	3.55160000	0.67716100
H	-5.03899500	1.05949700	-2.32635900
H	-4.74203500	3.29448700	-1.28374000
C	0.34295100	-2.82448600	0.23083000
H	1.32061400	-2.99329200	-0.22385700
H	0.44379600	-2.92870500	1.31382100
H	-0.33234700	-3.60726900	-0.12368800
C	-0.51558300	-1.37285700	-1.66382000
H	0.39491400	-1.53806300	-2.24444100
H	-1.23913100	-2.14370000	-1.93828800
H	-0.92631700	-0.39931300	-1.94086500
H	4.92671200	-0.54526800	-1.25614300

(S,S)-**2**

E=-3283.230100 au
Charge = 0; Multiplicity = 1

C	2.13896600	1.50345100	0.95106400
C	4.09491200	1.03221500	-0.18760700
C	3.13152500	-0.13479900	-0.40720800
O	5.21208700	1.16326900	-0.64803800
O	1.32188200	2.19935600	1.52510900
N	1.97087200	0.23461600	0.44726900
N	3.45414300	1.89768300	0.65959400
H	3.83266600	2.78819400	0.96213500
C	3.80915700	-1.44695300	-0.00250000
H	3.14449100	-2.29515200	-0.16855800
H	4.70729900	-1.58802300	-0.60817900
H	4.09849400	-1.42813400	1.05110200
C	2.49703700	-0.18300600	-1.81398600
H	2.42911100	0.82903900	-2.22313300
H	3.07610500	-0.79603200	-2.50570600
C	1.09166900	-0.74699400	-1.54202200
H	1.12372600	-1.83679700	-1.51401500
H	0.38528100	-0.46036900	-2.31964700
C	0.66699200	-0.17209100	-0.16463600
H	0.08641300	0.74196300	-0.30733400
C	-0.17165600	-1.10314000	0.75314200
Se	-1.87676100	-1.61977300	-0.26016500
C	-2.89850000	0.03394100	-0.27907900
C	-3.88538500	0.24850200	0.68946400
C	-2.70624500	0.98051900	-1.29219200
C	-4.66542300	1.40529700	0.65018900
H	-4.04584200	-0.48477800	1.47078400
C	-3.48501900	2.13819100	-1.32383100
H	-1.95572900	0.81555100	-2.05590600
C	-4.46488100	2.35276000	-0.35375500
H	-5.42852200	1.56306600	1.40465900
H	-3.32840900	2.86828100	-2.11056800
H	-5.07112500	3.25145300	-0.38296900
C	-0.56089200	-0.40334800	2.05342200
H	-1.20318800	-1.04766200	2.65831000
H	0.33938600	-0.18110000	2.63469900
H	-1.07867000	0.53952600	1.87326500
C	0.48753300	-2.44954000	1.06144500
H	1.43487600	-2.28033500	1.58096800
H	-0.15219400	-3.04009200	1.72151300
H	0.69011000	-3.04343900	0.16811900

(S,R)-**2`**

E=-3283.223938 au
Charge = 0; Multiplicity = 1

C	-3.49962800	-0.77777400	0.36526600
C	-3.73988600	1.42445000	-0.30841600
C	-2.25803900	1.18846500	0.00258400
O	-4.24530500	2.45950400	-0.70279700
O	-3.90322500	-1.88813200	0.67001400
N	-2.23173200	-0.26980500	0.31343200
N	-4.37641500	0.25998300	-0.01583200
H	-5.37667400	0.11239800	-0.07997200
C	-1.91171000	2.08584900	1.20888500
H	-0.84798200	2.06279600	1.43919200
H	-2.18239400	3.11597300	0.96584000
H	-2.47013900	1.77914600	2.09623300
C	-1.37436600	1.48324500	-1.21611300
H	-1.83410100	1.04834200	-2.10952600
H	-1.31521000	2.56392200	-1.36555900
C	0.02399800	0.88984800	-1.02225700
H	0.53882800	1.38856000	-0.19771400
H	0.61538000	1.07416200	-1.92159900
C	-0.06400700	-0.61337300	-0.77571200
H	-0.50923400	-1.08141900	-1.65746700
C	-0.94375800	-1.04153700	0.44778700
Se	1.76968400	-1.47192500	-0.81505900
C	2.95168200	-0.09445500	-0.11117200
C	3.54038900	0.82730100	-0.98396300
C	3.29484800	-0.08065900	1.24482000
C	4.44939200	1.76844100	-0.49817100
H	3.29294200	0.80984900	-2.03856100
C	4.20543700	0.86155400	1.72560100
H	2.85610900	-0.80126100	1.92384800
C	4.78138100	1.78868100	0.85662500
H	4.89946000	2.48123300	-1.18073300
H	4.46540200	0.86672000	2.77865100
H	5.48958300	2.51907700	1.23227900
C	-1.19825600	-2.55373100	0.34783100
H	-0.22820100	-3.05466000	0.33287600
H	-1.76232100	-2.92571700	1.20040200
H	-1.73370600	-2.81622800	-0.56615800
C	-0.29460100	-0.74558400	1.81084100
H	-1.02497200	-0.90294400	2.60830200

H	0.53718900	-1.43191000	1.97580600
H	0.08878100	0.27086500	1.88831400

(S,S)-**2`**

E=-3283.226455 au
Charge = 0; Multiplicity = 1

C	-3.64855900	-1.54346000	0.25560200
H	-4.14051100	-1.19622100	1.16689700
H	-4.40960700	-1.93400500	-0.42405400
H	-2.97474600	-2.36016900	0.50856200
C	-2.90860600	-0.38619700	-0.44649300
C	-2.05356300	-0.86933500	-1.62454700
C	-0.77066700	-1.53755900	-1.12270000
H	-1.79218600	-0.01236700	-2.25409400
H	-2.63696100	-1.56342400	-2.23394000
C	-0.75014100	-0.02093700	1.00257000
C	0.02950900	-0.57759400	-0.23897800
H	-0.15873900	-1.82421700	-1.98033800
H	-1.00362100	-2.45855300	-0.57949400
C	-0.96530900	-1.06814700	2.11266900
H	-0.02257700	-1.25911600	2.62814600
H	-1.67899900	-0.68556000	2.84621600
H	-1.33681100	-2.02016200	1.73457800
C	0.05096300	1.15016400	1.58997200
H	-0.41911600	1.55195600	2.48506500
H	1.03854100	0.77310800	1.85989000
H	0.17768100	1.95959600	0.86925100
H	0.34171000	0.27796200	-0.83956100
Se	1.75934000	-1.48416900	0.25716900
C	3.06155500	-0.11796200	-0.20042900
C	4.08906200	0.14659300	0.71074400
C	3.03867500	0.54387800	-1.43261700
C	5.08727400	1.06840700	0.38947600
H	4.10794100	-0.35875700	1.66968100
C	4.02664000	1.48018300	-1.73770800
H	2.25909000	0.33098200	-2.15469400
C	5.05571300	1.74196600	-0.83122100
H	5.88133300	1.26615900	1.10145100
H	3.99784800	1.99496300	-2.69210300
H	5.82646800	2.46441700	-1.07573400
C	-3.97890900	0.60933000	-0.90598300
N	-3.78562500	1.73658400	-0.17214100
H	-4.36917400	2.56364700	-0.21393000

N	-2.08261700	0.44563100	0.47580700
C	-2.66315200	1.66637600	0.67971700
O	-2.35695300	2.58215200	1.42545400
O	-4.84684200	0.41307000	-1.73690800

(S,R)-**INT4**

E=-3244.349032 au
Charge = 1; Multiplicity = 1

C	-3.33064200	0.58154300	-0.93808500
C	-3.05837600	-0.48303600	1.09809300
C	-2.21531100	-1.24985200	0.05805900
O	-3.17003300	-0.74719600	2.27831300
O	-3.77786300	1.37611100	-1.74725200
N	-2.44418100	-0.42676300	-1.13527100
H	-2.20464000	-0.72871800	-2.06969800
N	-3.65413300	0.55306900	0.43031500
C	-2.77696400	-2.66872000	-0.11704400
H	-2.28978700	-3.17407600	-0.95318300
H	-2.61013400	-3.25463400	0.78925600
H	-3.84987700	-2.63346600	-0.32056700
C	-0.73685700	-1.22452200	0.51128000
H	-0.45598100	-0.18602800	0.69829700
H	-0.68021600	-1.75784200	1.46381800
C	0.22351900	-1.85898600	-0.49803800
H	0.17169500	-1.35648600	-1.46881600
H	-0.07347000	-2.89597700	-0.69327200
C	1.67347000	-1.97148000	-0.08427100
C	2.18466200	-1.47288800	1.17732600
H	-4.28241400	1.22421700	0.85598200
Se	2.90855800	-0.11186300	-0.14956300
C	1.69772900	1.38504200	0.03195300
C	1.12954100	1.90434300	-1.13295600
C	1.51031500	2.00308800	1.27073200
C	0.34858600	3.05722400	-1.04952800
H	1.29162400	1.41940400	-2.08816400
C	0.72070000	3.14878100	1.34003400
H	1.96776700	1.59701100	2.16490000
C	0.14169600	3.67521200	0.18330500
H	-0.09852800	3.46687800	-1.94811800
H	0.56449800	3.63392600	2.29689300
H	-0.46850400	4.56931100	0.24402500
C	2.47054400	-3.01725000	-0.81093900
H	3.54280200	-2.93162400	-0.62802400

H	2.13980700	-3.99101100	-0.42673600
H	2.27398900	-2.99533100	-1.88392400
H	1.51259100	-0.98967700	1.87545400
H	3.03347800	-1.97900800	1.62371400

(S,S)-**INT4**

E=-3244.354071 au
Charge = 1; Multiplicity = 1

C	-4.21032700	0.47899300	-1.43226200
C	-3.81504700	1.20387700	0.72909200
C	-3.18545000	-0.19826400	0.58855700
O	-3.81525800	1.90248800	1.72246000
O	-4.66118800	0.49298000	-2.56489900
N	-3.46538500	-0.47882700	-0.82499100
H	-3.37575400	-1.39897900	-1.23317200
N	-4.37074800	1.50610400	-0.48532800
H	-4.86947000	2.36396400	-0.68977200
C	-3.90531400	-1.19197900	1.51241900
H	-3.56765000	-2.21044500	1.30931700
H	-3.69570600	-0.95818200	2.55835100
H	-4.98540200	-1.15280100	1.35202500
C	-1.67075300	-0.08648400	0.87201600
H	-1.25133800	0.67224900	0.20397800
H	-1.55628200	0.27619900	1.89551700
C	-0.90597800	-1.40028800	0.68160100
H	-1.12136000	-1.83837300	-0.29706900
H	-1.22570000	-2.13851300	1.42969900
C	0.59087400	-1.30686200	0.86742200
C	1.42976500	-2.25476100	0.15335500
Se	1.49520100	-0.58510300	-0.99368700
C	3.12368700	0.26269800	-0.37962800
C	4.34194200	-0.41784300	-0.42374500
C	3.05363100	1.60501100	-0.00141800
C	5.50570200	0.25827900	-0.06159200
H	4.38370400	-1.45576900	-0.73109800
C	4.22735400	2.27074400	0.35142900
H	2.10184400	2.12205200	0.02039500
C	5.44907500	1.59860500	0.32470700
H	6.45623000	-0.26234100	-0.08735600
H	4.18260500	3.31188600	0.65009600
H	6.35853100	2.11958700	0.60213400
C	1.12219500	-0.63856100	2.09941700
H	0.75740500	-1.22521400	2.95292500

H	2.21078500	-0.63919900	2.13279300
H	0.75055000	0.37933400	2.21825100
H	2.41238200	-2.48700000	0.54784800
H	0.95415500	-3.03863600	-0.42597800

(S,R)-**INT5**

E= -3244.347842 au
Charge = 1; Multiplicity = 1

C	2.31189200	1.68092600	-0.33994100
C	4.07533800	0.17926200	-0.43203700
C	3.26617000	-0.26542200	0.79360800
O	5.08982100	-0.31858400	-0.84199800
O	1.63471200	2.65342000	-0.47996700
N	2.00424400	0.60215100	0.66345900
N	3.45464600	1.30225000	-0.96822500
C	4.04242500	0.06473500	2.06785900
H	3.41828200	-0.13951200	2.93990200
H	4.92639400	-0.57364500	2.11529600
H	4.36298300	1.10887400	2.08740300
C	2.77693500	-1.71533100	0.69386900
H	3.53918300	-2.34225900	0.23085500
H	2.59332300	-2.09565900	1.69970200
C	1.48750400	-1.64900100	-0.12362900
H	0.85845400	-2.52526100	0.03047700
H	1.71200100	-1.57410400	-1.19133300
C	0.74818700	-0.38562400	0.34069700
C	-0.13800200	0.24529700	-0.73211600
Se	-1.66884300	-0.91182700	-1.26929700
C	-3.10233200	-0.10691200	-0.23157800
C	-3.88978500	-0.92728100	0.58061200
C	-3.40098500	1.25427400	-0.34996900
C	-4.97213400	-0.38319500	1.27591500
H	-3.65944000	-1.98206800	0.67649200
C	-4.47057700	1.79433300	0.36313700
H	-2.80675100	1.89061000	-0.99568500
C	-5.26051700	0.97711100	1.17411500
H	-5.58067100	-1.02412500	1.90459900
H	-4.69431400	2.85182500	0.27267800
H	-6.09645600	1.39934800	1.72069000
H	3.85426400	1.85106900	-1.72622500
H	1.78807100	1.07843200	1.54607300
C	0.04263300	-0.55030600	1.68327700
H	-0.37833800	0.40056900	2.01973700

H	-0.77067100	-1.26714700	1.56731300
H	0.71940200	-0.92849700	2.45148600
H	-0.56739900	1.19014700	-0.40827600
H	0.41336200	0.39681600	-1.65892700

(S,S)-**INT5**

E= -3244.348452 au
Charge = 1; Multiplicity = 1

C	-2.25717400	1.63012500	-0.74016700
C	-4.02971500	0.85809900	0.55110400
C	-3.32237100	-0.41774000	0.09867700
O	-5.01494400	0.93158800	1.23637000
O	-1.52666800	2.32309500	-1.38105100
N	-2.09416500	0.13888700	-0.63863400
H	-2.06944900	-0.22551800	-1.59818400
N	-3.34353700	1.94376200	0.01097200
H	-3.64722200	2.90772500	0.13005100
C	-4.20470000	-1.21560700	-0.85983100
H	-3.67271600	-2.10972100	-1.19127600
H	-5.10810600	-1.52234500	-0.33063100
H	-4.49114900	-0.62025400	-1.73027600
C	-2.73915900	-1.25887900	1.25457000
H	-2.68940800	-0.67437900	2.17416100
H	-3.36735900	-2.12937000	1.43813200
C	-1.33818300	-1.64283300	0.77054300
H	-1.39657500	-2.45507200	0.04153000
H	-0.69361400	-1.96260500	1.58817500
C	-0.76325900	-0.38654600	0.09615600
C	0.24560800	-0.65987800	-1.02152000
Se	1.88030300	-1.62779500	-0.44498300
C	3.07884400	-0.15420000	-0.02700500
C	3.33627300	0.86252200	-0.95156900
C	3.75416800	-0.17355400	1.19653600
C	4.24922000	1.86926200	-0.63891000
H	2.83134800	0.87180800	-1.91069100
C	4.68349200	0.82570100	1.49348800
H	3.55443300	-0.95882600	1.91644600
C	4.92737500	1.85173300	0.58146500
H	4.43913300	2.66017800	-1.35640100
H	5.20611400	0.80434600	2.44360200
H	5.64225600	2.63207900	0.81754100
C	-0.28683600	0.65625000	1.10239200
H	0.53347300	0.21514200	1.67015300

H	0.09006700	1.55503600	0.61307400
H	-1.07151800	0.93329100	1.80893700
H	-0.19224300	-1.31966800	-1.77313400
H	0.56756500	0.26044400	-1.50626500

(S,R)-**INT5`**

E= -3244.340727 au
Charge = 1; Multiplicity = 1

C	3.18201700	1.27873000	-0.73227800
C	3.95962700	-0.90882700	-0.62231000
C	2.79720500	-0.88822600	0.37532200
O	4.66214900	-1.84546400	-0.89194300
O	2.94752800	2.39662600	-1.06726100
N	2.54048300	0.62756200	0.48944000
N	4.02828300	0.34614000	-1.22961500
H	4.64771400	0.55123100	-2.01071600
C	3.17590900	-1.46862100	1.73232400
H	2.32402000	-1.44108100	2.41316500
H	3.47439900	-2.50843400	1.59379600
H	4.01096600	-0.92261700	2.17909100
C	1.59150600	-1.59673100	-0.29082300
H	1.60975300	-1.42551700	-1.36961300
H	1.73464900	-2.66708200	-0.13451800
C	0.23799300	-1.15445400	0.27980400
H	0.17466600	-1.41663100	1.33827500
H	-0.54833100	-1.70273900	-0.24216100
C	0.02290800	0.35590100	0.11148000
C	1.13443500	1.10646900	0.84881100
Se	-1.63221000	0.93727300	1.16952000
C	-3.03589100	0.06419900	0.15167900
C	-3.71247800	0.76574200	-0.85218000
C	-3.41727300	-1.24476900	0.46625400
C	-4.75532300	0.15195100	-1.54739500
H	-3.42781800	1.78354700	-1.09039100
C	-4.45862600	-1.85371700	-0.23503200
H	-2.90784100	-1.78459800	1.25506900
C	-5.12701400	-1.15767300	-1.24266300
H	-5.27600300	0.69934700	-2.32554900
H	-4.74817500	-2.86967000	0.01053600
H	-5.93766000	-1.63227500	-1.78454100
H	3.12689800	0.96171100	1.26716900
C	-0.13981500	0.79371300	-1.34526700
H	-0.99769900	0.29507200	-1.79988500

H	-0.28569700	1.87291500	-1.41750000
H	0.73391200	0.53010100	-1.94970300
H	1.12892200	2.17663600	0.64757200
H	1.06859600	0.94546300	1.92473800

(S,S)-**INT5`**

E=-3244.342625 au
Charge = 1; Multiplicity = 1

C	-2.96214700	-0.11988600	1.43640400
C	-2.71340200	-1.72299800	-0.22455200
C	-2.23627000	-0.43152700	-0.89290700
O	-2.76226300	-2.81621600	-0.72160200
O	-3.26252600	0.46286000	2.43189300
N	-2.21902800	0.54637800	0.29706100
H	-2.73114100	1.40342200	0.05661800
N	-3.13090000	-1.41140600	1.06778600
C	-3.29398900	0.02900300	-1.90103000
H	-2.99745000	0.97610200	-2.35450600
H	-3.38301600	-0.72194300	-2.68708300
H	-4.26898200	0.15307300	-1.42328700
C	-0.82602000	-0.56650300	-1.49306400
H	-0.20963000	-1.20427400	-0.85650900
H	-0.92893800	-1.07167200	-2.45453200
C	-0.16077200	0.80055000	-1.68351700
H	-0.76118100	1.40384900	-2.37036900
H	0.80546900	0.65985600	-2.16737700
C	-0.00961400	1.58987800	-0.35874300
C	-0.81119800	0.94197600	0.77368500
Se	1.91228000	1.65667500	0.31859800
C	2.48101200	-0.19763300	0.21995300
C	2.32269600	-1.04181300	1.32517400
C	3.11966600	-0.67028200	-0.93191500
C	2.78129900	-2.35820700	1.26735600
H	1.84847000	-0.67233500	2.22655500
C	3.57804600	-1.98749300	-0.98258600
H	3.26223400	-0.01387100	-1.78186200
C	3.40616300	-2.83301800	0.11370900
H	2.65410400	-3.00882400	2.12571800
H	4.07204700	-2.34942900	-1.87770400
H	3.76432200	-3.85572600	0.07211200
H	-3.56087000	-2.09140700	1.69081800
C	-0.37430100	3.06654600	-0.53895600
H	0.20573700	3.50809300	-1.35211300

H	-1.43435500	3.16609500	-0.79792300
H	-0.19407400	3.64141100	0.37333000
H	-0.36172300	0.01659800	1.12740200
H	-0.95773700	1.62453600	1.60804900

(S,R)-**4**

E=-3243.929453 au
Charge = 0; Multiplicity = 1

C	-2.44938700	-1.57070200	-0.27673900
C	-4.31198800	-0.20779200	-0.07978000
C	-3.17700000	0.42751400	0.73153900
O	-5.43470900	0.22505900	-0.24807000
O	-1.80510000	-2.52699300	-0.66964700
N	-2.02949800	-0.48047300	0.45179200
N	-3.82711600	-1.41501900	-0.50792000
C	-3.61214300	0.38999000	2.20734300
H	-2.80279400	0.74015200	2.84894100
H	-4.47507900	1.04580300	2.34888500
H	-3.89072600	-0.62212300	2.51255800
C	-2.71495200	1.83175200	0.27631100
H	-3.45449000	2.30540100	-0.37112800
H	-2.59090800	2.46938500	1.15346700
C	-1.37380600	1.60502100	-0.43925000
H	-0.70125700	2.45701700	-0.33544800
H	-1.53872900	1.43381400	-1.50685500
C	-0.77080500	0.31735900	0.18097900
C	0.15305600	-0.41985500	-0.80046900
Se	1.72807500	0.63278800	-1.41696000
C	3.12103900	0.03426600	-0.20124200
C	3.96635400	0.99714600	0.35906300
C	3.34506700	-1.32159700	0.05772700
C	5.02866800	0.60375900	1.17527900
H	3.79363500	2.04992500	0.16616100
C	4.39497100	-1.70628600	0.89153200
H	2.71059200	-2.07832800	-0.38873500
C	5.24202700	-0.74682800	1.44933900
H	5.67997900	1.35725900	1.60498500
H	4.55777900	-2.75963600	1.09339800
H	6.06176300	-1.05061600	2.09087400
H	-4.36861800	-2.10453500	-1.01652300
H	-0.36794500	-0.64880200	-1.72765800
H	0.53691000	-1.34715000	-0.38401100
C	-0.06486800	0.58988000	1.51475200

H	0.23103300	-0.34885400	1.99007700
H	0.83042300	1.19213900	1.34875600
H	-0.70874700	1.13650900	2.20520400

(S,S)-**4**

E=-3243.935045 au
Charge = 0; Multiplicity = 1

C	-2.33222500	1.72488400	-0.29774900
C	-4.32836100	0.63287400	0.15999700
C	-3.24227400	-0.42774400	-0.02474300
O	-5.49986200	0.45494100	0.43217800
O	-1.58664000	2.67731400	-0.44791300
N	-2.03181100	0.39057200	-0.26851700
N	-3.72368200	1.83389000	-0.10729200
H	-4.19781400	2.72983800	-0.09834700
C	-3.60987800	-1.30444200	-1.23260900
H	-2.83238700	-2.04818100	-1.41567900
H	-4.55000400	-1.82593900	-1.03785500
H	-3.72687600	-0.69530200	-2.13240600
C	-2.84924100	-1.24744700	1.21559000
H	-2.98684600	-0.65015000	2.12070000
H	-3.43679700	-2.16121600	1.31112900
C	-1.35631700	-1.52002100	0.96319200
H	-1.24111300	-2.36116300	0.27551700
H	-0.81997100	-1.76758900	1.87909000
C	-0.78721600	-0.22045600	0.31616100
C	0.17079200	-0.50460700	-0.85508600
Se	1.78942000	-1.57892600	-0.41511300
C	3.11105600	-0.18550300	-0.11540000
C	3.33903600	0.81659700	-1.06408900
C	3.89909400	-0.24184100	1.03822500
C	4.33509800	1.76810200	-0.84623600
H	2.74787900	0.85585800	-1.97177900
C	4.90838000	0.70206200	1.24068900
H	3.72391600	-1.01314000	1.77956500
C	5.12503400	1.71170900	0.30356300
H	4.50155900	2.54553100	-1.58413500
H	5.51545200	0.65124500	2.13813300
H	5.90404700	2.44838400	0.46543400
C	-0.15757700	0.68369900	1.38185700
H	0.69425000	0.16823600	1.82898200
H	0.18873700	1.62808300	0.96215300
H	-0.87666600	0.89748900	2.17715700

H	-0.34015400	-1.09521000	-1.61530200
H	0.52057300	0.42024500	-1.31215300

(S,R)-**4`**

E= -3243.942165 au
Charge = 0; Multiplicity = 1

C	-3.05859400	-0.41509700	1.30559500
C	-3.73500900	1.10397000	-0.30755100
C	-2.20332500	1.01938100	-0.36100700
O	-4.45346900	1.79098900	-1.00837600
O	-3.17376100	-1.24858900	2.19073200
N	-1.92877700	0.04874400	0.71427500
N	-4.13249100	0.26397100	0.69662700
H	-5.09591900	0.11325300	0.97146300
C	-1.60189200	2.39965300	-0.05674800
H	-0.51149900	2.35573300	-0.04865600
H	-1.91153200	3.11150900	-0.82467900
H	-1.94076600	2.76324300	0.91646000
C	-1.71433900	0.43179700	-1.70454900
H	-2.39202600	-0.36843100	-2.01437100
H	-1.75503000	1.20766700	-2.47255600
C	-0.27539500	-0.11297000	-1.59222300
H	0.41000600	0.72089600	-1.42404800
H	0.00579400	-0.57291500	-2.54300600
C	-0.12165300	-1.14159800	-0.45862400
C	-0.59825600	-0.53237300	0.87443800
Se	1.82572800	-1.69728400	-0.25349400
C	2.74244200	-0.01895700	0.09404900
C	3.22445600	0.75562500	-0.96741700
C	2.98731000	0.38280400	1.41230700
C	3.92953400	1.93207900	-0.70936300
H	3.05391400	0.44132500	-1.99009800
C	3.69631700	1.55811300	1.66460100
H	2.62840900	-0.21997100	2.23805100
C	4.16516100	2.33549900	0.60526600
H	4.29816800	2.52883600	-1.53670700
H	3.88194700	1.86294200	2.68886100
H	4.71652600	3.24821500	0.80310300
H	-0.65266000	-1.28801800	1.65727300
H	0.08802700	0.25247000	1.19945600
C	-0.83409700	-2.46274200	-0.76592700
H	-0.50496200	-2.87269600	-1.72430300
H	-0.64716600	-3.20602300	0.01391700

H	-1.91523700	-2.30968800	-0.82360300

(S,S)-**4`**

E=-3243.941172 au
Charge = 0; Multiplicity = 1

C	3.10495900	-1.26187300	-1.74717400
H	3.89985700	-0.63370800	-2.15679900
H	3.43653000	-2.30218500	-1.76168100
H	2.22741900	-1.17244900	-2.38972900
C	2.77533000	-0.84548000	-0.30568000
C	1.61704900	-1.66219700	0.31014700
C	0.25337900	-1.20369600	-0.24501300
H	1.63726100	-1.54688800	1.39707200
H	1.76720400	-2.72195200	0.09185500
C	1.17242400	1.10434300	-0.67733500
C	0.01205800	0.30479100	-0.04231900
H	-0.53802800	-1.77245900	0.24910500
H	0.19855600	-1.43762600	-1.31102300
Se	-1.60141100	0.90481900	-1.13726500
C	-3.06885600	0.08587900	-0.16447400
C	-3.79498900	0.83897900	0.76468700
C	-3.44731600	-1.23377600	-0.43572100
C	-4.88476300	0.27006900	1.42567100
H	-3.51264500	1.86448200	0.97102900
C	-4.53418400	-1.79934100	0.23246100
H	-2.90039800	-1.81619200	-1.16720800
C	-5.25379300	-1.04938800	1.16340500
H	-5.44331400	0.86006000	2.14425800
H	-4.82036600	-2.82365600	0.01942000
H	-6.10084600	-1.48973000	1.67787300
C	4.04271000	-0.92896600	0.55551900
N	4.29126700	0.34127800	0.99953100
H	5.07174000	0.60203200	1.59049900
N	2.45249100	0.59007500	-0.20824500
C	3.32033700	1.27205700	0.58258100
O	3.30556400	2.45155800	0.89732200
O	4.70318000	-1.92305600	0.78891700
H	1.11648400	2.15956400	-0.41148200
H	1.13737000	1.02281000	-1.76819700
C	-0.19467700	0.67523800	1.42522400
H	-0.38683500	1.74426600	1.54091300
H	-1.03162500	0.12235000	1.85720200
H	0.69535000	0.43010500	2.01312100
